# Anaplasma phagocytophilum Hijacks Flotillin and NPC1 Complex To Acquire Intracellular Cholesterol for Proliferation, Which Can Be Inhibited with Ezetimibe

**DOI:** 10.1128/mBio.02299-21

**Published:** 2021-09-21

**Authors:** Weiyan Huang, Qingming Xiong, Mingqun Lin, Yasuko Rikihisa

**Affiliations:** a Department of Veterinary Biosciences, The Ohio State University, Columbus, Ohio, USA; Yale University School of Medicine

**Keywords:** flotillin, NPC1, cholesterol, ezetimibe, intracellular cholesterol transport, *Anaplasma*, intracellular bacteria

## Abstract

The intracellular cholesterol transport protein Niemann-Pick type C1 (NPC1) and lipid-raft protein flotillin (FLOT) are required for cholesterol uptake by the obligatory intracellular bacterium Anaplasma phagocytophilum and for infection, and each protein localizes to membrane-bound inclusions containing replicating bacteria. Here, we found striking localization of FLOT2 in NPC1-lined vesicles and a physical interaction between FLOT2 and NPC1. This interaction was cholesterol dependent, as a CRAC (cholesterol recognition/interaction amino acid cholesterol-binding) domain mutant of FLOT2 did not interact with NPC1, and the cholesterol-sequestering agent methyl-β-cyclodextrin reduced the interaction. The stomatin-prohibitin-flotillin-HflC/K domain of FLOT2, FLOT2^1–183^, was sufficient for the unique FLOT2 localization and interaction with NPC1. NPC1, FLOT2, and FLOT2^1–183^ trafficked to the lumen of *Anaplasma* inclusions. A loss-of-function mutant, NPC1^P691S^ (mutation in the sterol-sensing domain), did not colocalize or interact with FLOT2 or with *Anaplasma* inclusions and inhibited infection. Ezetimibe is a drug that blocks cholesterol absorption in the small intestine by inhibiting plasma membrane Niemann-Pick C1-like 1 interaction with FLOTs. Ezetimibe blocked the interaction between NPC1 and FLOT2 and inhibited *Anaplasma* infection. Ezetimibe did not directly inhibit *Anaplasma* proliferation but inhibited host membrane lipid and cholesterol traffic to the bacteria in the inclusion. These data suggest that *Anaplasma* hijacks NPC1 vesicles containing cholesterol bound to FLOT2 to deliver cholesterol into *Anaplasma* inclusions to assimilate cholesterol for its proliferation. These results provide insights into mechanisms of intracellular cholesterol transport and a potential approach to inhibit *Anaplasma* infection by blocking cholesterol delivery into the lumen of bacterial inclusions.

## INTRODUCTION

The Gram-negative obligatory intracellular bacterium Anaplasma phagocytophilum primarily infects granulocytes and causes the emerging tick-borne zoonosis called human granulocytic anaplasmosis (HGA). The HGA cases reported to the CDC have increased greater than 10-fold during the past 10 years, reaching nearly 6,000 in 2017 (1). Early clinical signs of HGA are mild to moderate, including fever, chills, severe headache, muscle aches, nausea, vomiting, diarrhea, and loss of appetite, which are readily resolved in most cases with appropriate treatment. However, if treatment is delayed or if there are other medical conditions present, HGA can cause severe illness requiring hospitalization in 36% of cases, and life-threatening disease occurs in 3% with the case fatality rate at 0.6% (2). The only effective treatment is the broad-spectrum antibiotic doxycycline, and there is no vaccine.

Unlike most Gram-negative bacteria, A. phagocytophilum lacks lipopolysaccharide and peptidoglycan in its membrane, yet it contains a significant amount of membrane cholesterol (free cholesterol, not cholesterol esters or lipid droplets) to support its membrane structure and functions (3). Cholesterol is essential for this bacterium, and mice with high blood cholesterol develop more severe clinical signs with a 10-fold higher bacterial load in the blood than mice with a normal cholesterol level (4). A. phagocytophilum cannot synthesize or modify cholesterol; thus, it must acquire cholesterol from its host cell (3, 5). Indeed, unlike most bacteria, A. phagocytophilum can readily take up exogenous cholesterol (3).

Mammalian cells acquire cholesterol from two sources: serum lipoproteins and via biosynthesis at the endoplasmic reticulum. A. phagocytophilum captures host cholesterol derived exclusively from low-density lipoprotein (LDL) by upregulating the cellular level of LDL receptor and subverting the Niemann-Pick type C1 (NPC1) pathway of cholesterol transport to A. phagocytophilum-containing inclusions (5, 6). In acidic endosomes, cholesterol esters in LDL are hydrolyzed by acid lipase to liberate free cholesterol, which enters intracellular vesicles containing the cholesterol-binding transmembrane protein, NPC1; NPC1-containing vesicles then transport cholesterol to the *trans*-Golgi network before cholesterol is distributed to various cellular destinations (7, 8). Certain mutations in the NPC1 gene causes Niemann-Pick Type C disease, owing to a defect in the trafficking of endocytosed cholesterol with sequestration of free cholesterol in lysosomes and late endosomes (9). Garver et al. (10) pointed out that NPC1 is found in two morphologically distinct membrane compartments, namely, large vesicles (diameter, ∼0.4 μm) that contain extensive internal membranes and caveolin-1 but lack lysosomal-associated membrane protein 1 (LAMP1), and a smaller diffusely distributed LAMP1-positive compartment. Only large NPC1-bearing vesicles devoid of lysosomal markers were found to be increased in the human promyelocytic leukemia cell line HL-60 infected with A. phagocytophilum, and this subset trafficked to the bacterial inclusions (6). This localization was abolished by the LDL-derived cholesterol-trafficking inhibitor U18666A, which, when administered to cells, mimics the molecular aspects of Niemann-Pick Type C disease (6). Studies using an NPC1-specific short interfering RNA (siRNA) and a cell line with dysfunctional NPC1 demonstrated that NPC1 function is required for cholesterol acquisition by A. phagocytophilum and infection (6).

Flotillin 1 (FLOT1) and FLOT2 are cholesterol-associated lipid-raft proteins that form a heterodimer and/or oligomer complex, and they are found in the plasma membrane, intracellular vesicles devoid of LAMP1, and exosomes (11 to 15). FLOTs are crucial for A. phagocytophilum replication in host cells, as siRNA-mediated knockdown of either FLOT1 or FLOT2 reduced A. phagocytophilum infection (16). FLOT-containing vesicles are enriched with free cholesterol and colocalize with endocytosed LDL and with acid lipase (16), and traffic to A. phagocytophilum inclusions (16). However, the relationship between NPC1 and FLOTs in uninfected or A. phagocytophilum*-*infected cells remains unknown, except for one study showing that the FLOT2-dependent release of cholesterol from exosomes ameliorates cellular cholesterol accumulation in Niemann-Pick type C disease (17). We investigated the interaction between NPC1 and FLOTs in the context of cholesterol transport within uninfected and A. phagocytophilum*-*infected host cells and explored the possibility of inhibiting this interaction to block A. phagocytophilum infection.

## RESULTS

### Lipid raft protein FLOT2 localizes to the lumen of NPC1-containing vesicles and interacts physically with NPC1.

**FIG 1:**
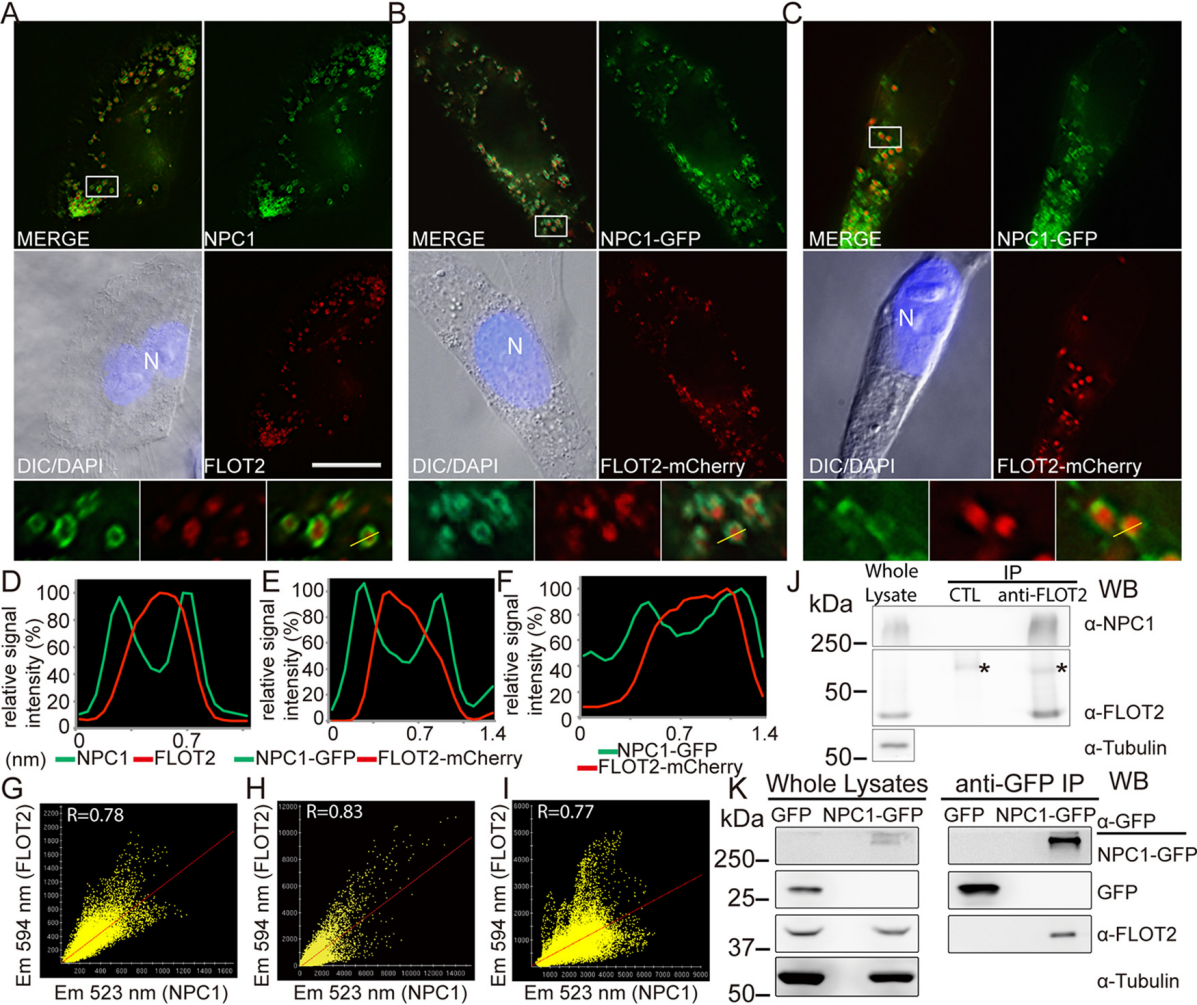
FLOT 2 localizes to the lumen of NPC1 vesicles and physically interacts with NPC1. (A to C) FLOT2 localizes inside NPC1-containing vesicles. (A) RF/6A cells were fixed, dually labeled with rabbit anti-NPC1 and mouse anti-FLOT2 antibodies, and stained with DAPI. (B and C) RF/6A cells (B) or HEK293T cells (C) were cotransfected with plasmids NPC1-GFP and FLOT2-mCherry, and at 2 dpt cells were fixed and stained with DAPI. DeltaVision deconvolution fluorescence microscopy images are representative of three independent experiments with similar results. N, nucleus. The boxed area is enlarged 4× in boxes at the bottom. Bar, 10 μm. DIC, differential interference contrast. (D to F) Relative signal intensity profiles for green (NPC or NPC1-GFP) and red (FLOT2 or FLOT2-mCherry) fluorescence from one selected vesicle each (as indicated by line drawing in the bottom right small box of panels A, B, and C) were normalized to the highest fluorescence intensity. (G to I) Colocalization of endogenous NPC1 and FLOT2 in RF/6A cells (G) and NPC1-GFP and FLOT2-mCherry in transfected RF/6A cells (H) and HEK293T cells (I) were analyzed, and the Pearson’s correlation coefficient was calculated using softWoRx. Em, emission. (J) Whole-cell lysates of HEK293T were immunoprecipitated (IP) with anti-FLOT2 or normal mouse IgG (CTL). (K) NPC1-GFP-transfected HEK293T cells at 3 dpt were IP with anti-GFP affinity gel. Lysates and immunoprecipitates were analyzed by Western blotting (WB) with antibodies against NPC1, FLOT2, GFP, and tubulin (loading control). *, mouse IgG heavy chain.

We first examined the topographical relationship between endogenous FLOT2 and endogenous NPC1-containing vesicles (here termed NPC1 vesicles) in thinly spread monkey endothelial RF/6A cells compatible for unambiguous localization analysis. By double immunofluorescence labeling, endogenous FLOT2 was distinctly localized in the lumen of endogenous NPC1-containing vesicles in RF/6A cells (Fig. 1A). Indeed, the fluorescence intensity profile analysis of red (FLOT2) and green (NPC1) signals along the length of the line revealed that the peak red signals were surrounded by peak green signals (Fig. 1D). Most of these NPC1 vesicles were found to be >0.4 μm in diameter. The majority of FLOT2 colocalized with NPC1 (Pearson’s correlation coefficient [*R* ]= 0.78) (Fig. 1G). To avoid possible artifacts of membrane permeabilization used for immunofluorescence labeling or immune cross-reactivity, we cotransfected RF/6A cells with FLOT2-mCherry and NPC1-GFP, which showed the same colocalization pattern as endogenous proteins (*R* = 0.83) (Fig. 1B, E, and H). This is not specific to RF/6A cells, as the colocalization pattern was observed with human kidney epithelial HEK293T cells (*R* = 0.77) (Fig. 1C, F, and I). Further, anti-FLOT2 IgG, but not negative-control mouse IgG, coimmunoprecipitated endogenous NPC1 in HEK293T cells (Fig. 1J). Immunoprecipitation with anti-GFP affinity gel resulted in pulldown of endogenous FLOT2 from the lysate of HEK293T cells transfected with NPC1-GFP but not cells transfected with the green fluorescent protein (GFP) negative control (Fig. 1K). These results demonstrate intraluminal localization of FLOT2 in NPC1 vesicles and physical interaction between FLOT2 and NPC1.

### The SPFH domain of FLOT2 is sufficient for interaction with NPC1.

**FIG 2:**
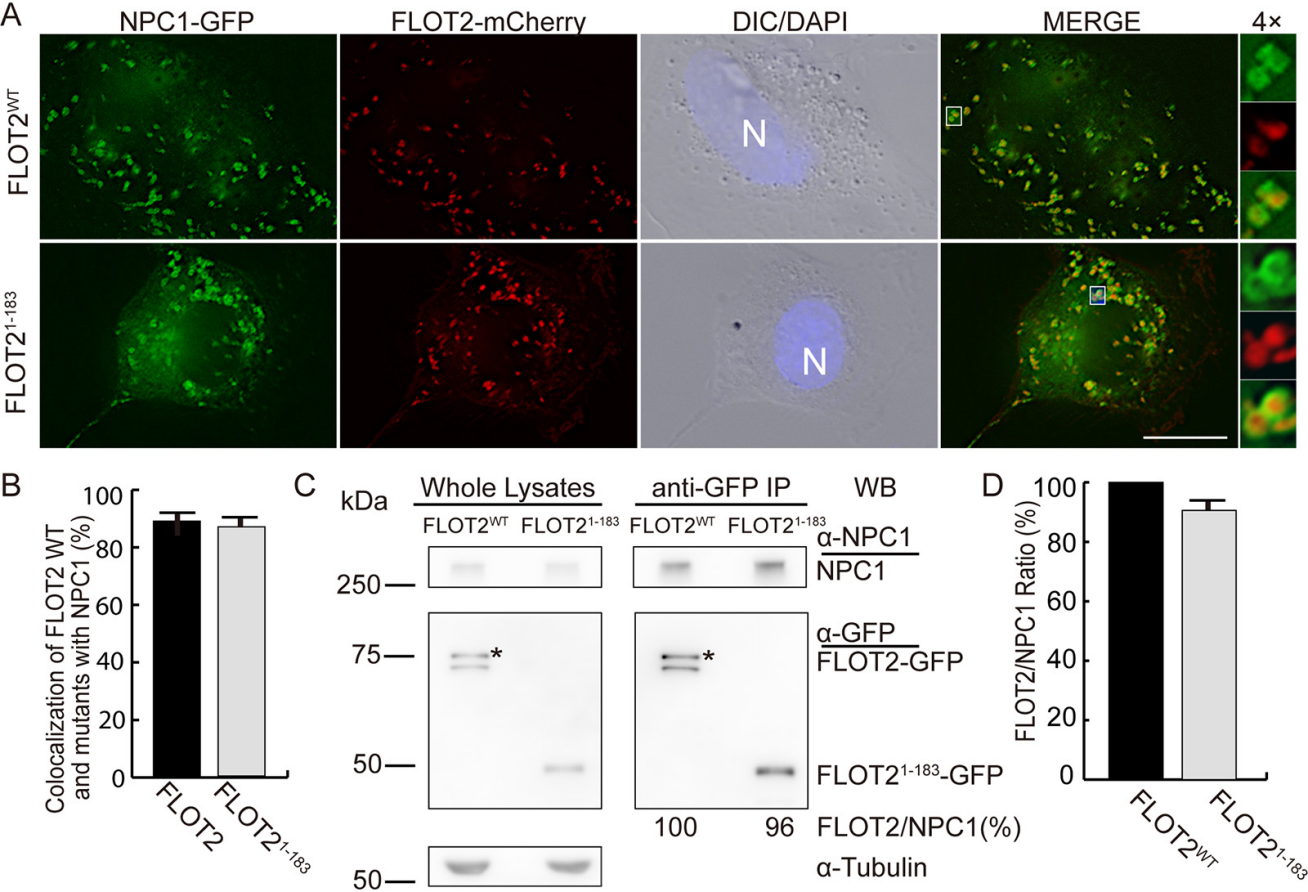
NPC1 colocalizes and interacts with the FLOT2 SPFH domain. (A) RF/6A cells were cotransfected with plasmids encoding NPC1-GFP and C-terminal mCherry-tagged FLOT2: wild type, FLOT2^WT^, or SPFH domain, FLOT2^1–183^. At 2 dpt, cells were fixed and stained with DAPI. Each boxed area is enlarged 4× on the right. Bar, 10 μm. (B) Colocalization of NPC1-GFP with FLOT2^WT^-mCherry or FLOT2^1–183^-mCherry was quantified by counting NPC1-GFP vesicles in 30 cells per group from three independent experiments. No significant difference was found between the WT and SPFH domain of FLOT2 by two-tailed *t* test. (C) Lysates of FLOT2^WT^-GFP- or FLOT2^1–183^-GFP-transfected HEK293T cells were immunoprecipitated (IP) with anti-GFP nanobody affinity gel and analyzed by Western blotting (WB) with anti-GFP and anti-NPC1. Asterisks indicate full-length FLOT2-GFP protein bands. (D) Relative ratios of WB band intensities (FLOT2/NPC1) were determined by ImageJ, with the ratio of FLOT2^WT^ set as 100%. The results are presented as the mean ± standard deviation from three independent experiments. No significant difference was found between the WT and SPFH domain of FLOT2 by two-tailed *t* test.

Human FLOT2 contains 428-amino-acid residues comprising an N-terminal SPFH (stomatin-prohibitin-flotillin-HflC/K) domain (residues 1 to 183), a FLOT domain comprising three coiled-coil domains (residues 184 to 363), and a C-terminal PDZ3 domain (residues 364 to 428) (18). To determine whether the SPFH domain is involved in the interaction with NPC1, we constructed two SPFH-only domain mutants, FLOT2^1–183^-mCherry and FLOT2^1–183^-GFP (Fig. 2A). Cotransfection of HEK293T cells with FLOT2^1–183^-mCherry and NPC1-GFP resulted in their colocalization in a manner similar to that observed with full-length FLOT2-mCherrry (Fig. 2A and B). Coimmunoprecipitation with anti-GFP resulted in pulldown of endogenous NPC1 from the lysate of HEK293T cells transfected with FLOT2-GFP or FLOT2^1–183^-GFP (Fig. 2C and D). These results indicated that FLOT2^1–183^ is sufficient for physical interaction with NPC1 and subsequent intraluminal localization in NPC1 vesicles.

### FLOT2 colocalization with NPC1 requires the FLOT2 cholesterol recognition motif.

**FIG 3:**
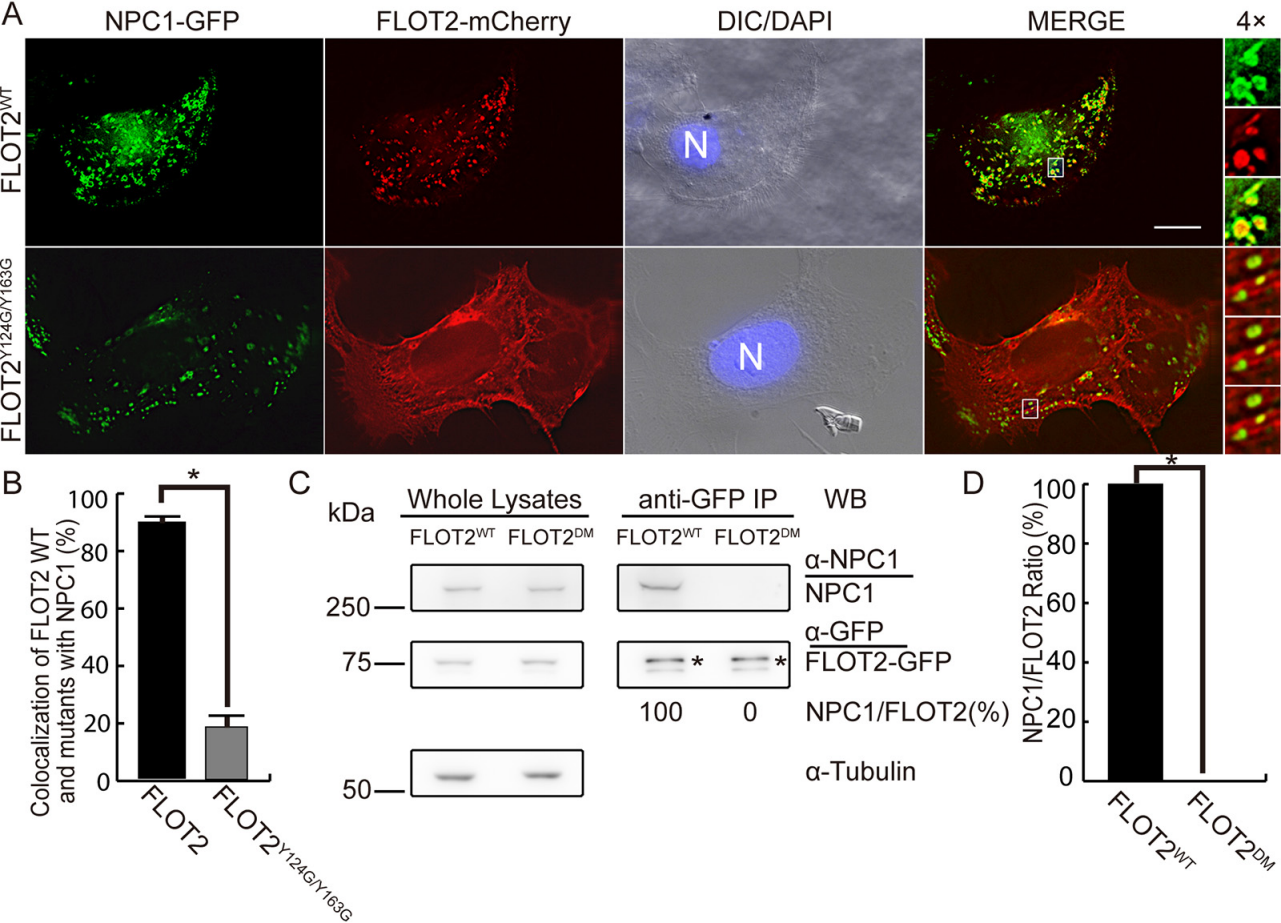
NPC1 does not colocalize or interact with the FLOT2 CRAC domain mutant. (A) RF/6A cells were cotransfected with plasmids encoding NPC1-GFP and C-terminal mCherry-tagged FLOT2: wild type, FLOT2^WT^, or double CRAC-motif mutant, FLOT2^Y124G/Y163G^. At 2 dpt, cells were fixed and stained with DAPI. Each boxed area is enlarged 4× on the right. Bar, 10 μm. (B) Colocalization of NPC1-GFP with FLOT2^WT^-mCherry or FLOT2^Y124G/Y163G^-mCherry was quantified by counting NPC1-GFP vesicles in 30 cells per group from three independent experiments. The results are presented as the mean ± standard deviation. *, *P < *0.05 by two-tailed *t* test. (C) Lysates of FLOT2^WT^-GFP- or FLOT2^Y124G/Y163G^-GFP-transfected HEK293T cells were immunoprecipitated (IP) with anti-GFP nanobody affinity gel and analyzed by Western blotting (WB) with anti-GFP and anti-NPC1. Asterisks indicate full-length FLOT2-GFP protein bands. (D) Relative ratios of WB band intensities (NPC1/FLOT2) were determined by ImageJ, with the ratio of FLOT2^WT^ set as 100%. The results are presented as the mean ± standard deviation from three independent experiments. *, *P < *0.05, significant difference between WT and CRAC-motif mutant FLOT2 by two-tailed *t* test.

FLOT2 has two cholesterol recognition/interaction amino acid cholesterol-binding motifs [CRAC; L/V-(X)(1-5)-Y-(X)(1-5)-R/K] within the SPFH domain (19). The CRAC motifs largely determine translocation of FLOT2 from the plasma membrane pool to the subcellular vesicular pool, as the double CRAC mutant FLOT2^Y124G/Y163G^-GFP is retained at the plasma membrane, whereas wild-type FLOT2-GFP localizes predominantly to vesicles (17). To determine whether the two CRAC motifs are required for NPC1 and FLOT2 colocalization, we cotransfected RF/6A cells with FLOT2^WT^- or FLOT2^Y124G/Y163G^-mCherry and NPC1-GFP (Fig. 3A and B). In agreement with previous findings with mouse oligodendroglial precursor cells (17) or RF/6A cells (16) transfected with FLOT2^Y124G/Y163G^-GFP or FLOT2^WT^-GFP, FLOT2^Y124G/Y163G^-mCherry in RF/6A cells localized mostly to the plasma membrane, whereas, FLOT2^WT^-mCherry localized to intracellular vesicles (Fig. 3A); consequently, colocalization of FLOT2^Y124G/Y163G^-mCherry with NPC1-GFP vesicles was significantly reduced (Fig. 3B). Coimmunoprecipitation with anti-GFP resulted in pulldown of endogenous NPC1 from the lysate of HEK293T cells transfected with FLOT2-GFP but not from the lysate of HEK293T cells transfected with FLOT2^Y124G/Y163G^-GFP (Fig. 3C and D). Thus, the CRAC motifs within the SPFH domain are required for the interaction and colocalization of NPC1 with FLOT2.

### FLOT2 colocalization with NPC1 requires the sterol-sensing domain of NPC1.

**FIG 4:**
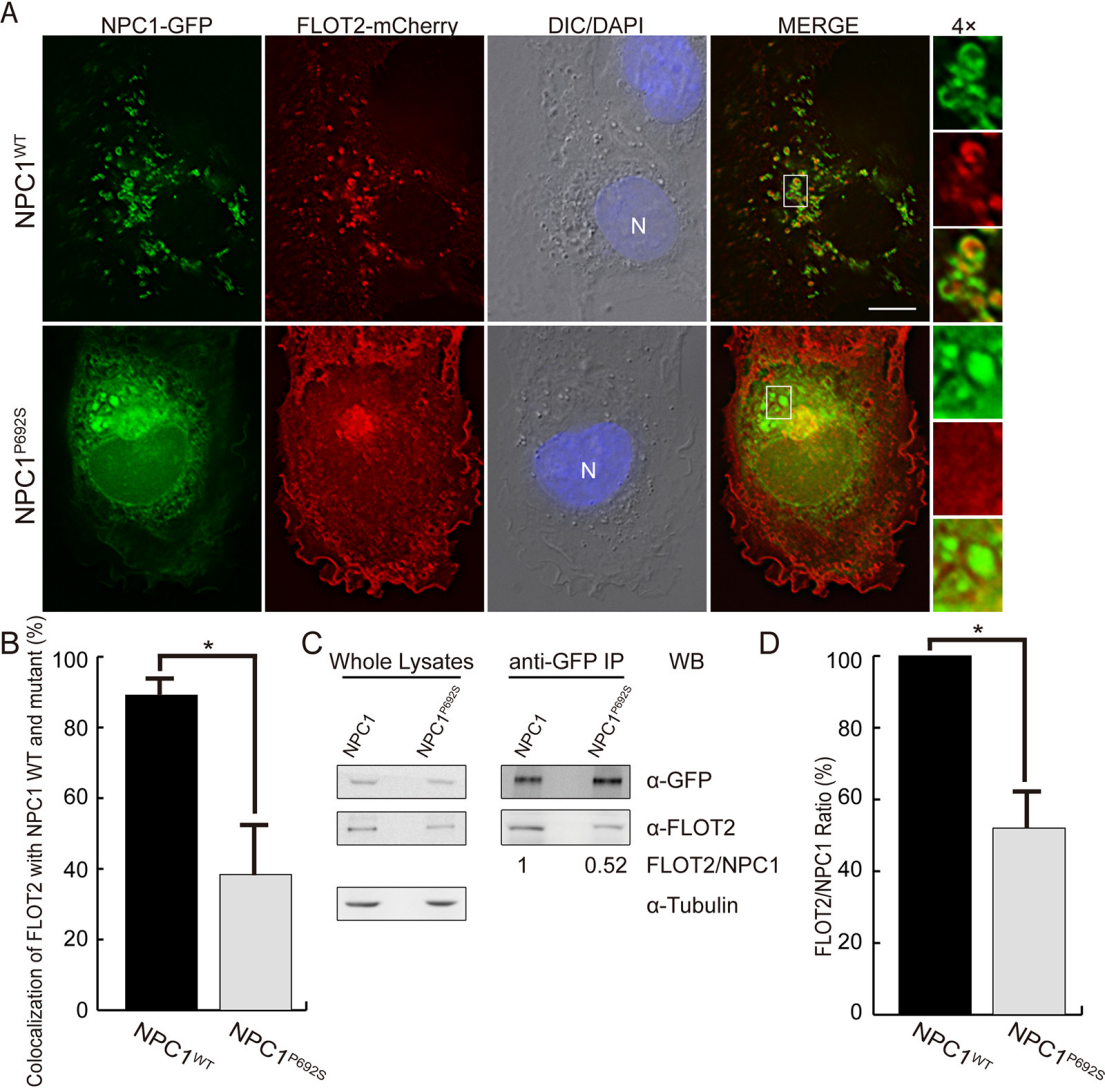
NPC1^P692S^ interacts with FLOT2 at reduced levels. (A) RF/6A cells were cotransfected with C-terminal GFP-tagged NPC1 wild type (NPC1^WT^) or P692S mutant (NPC1^P692S^) and FLOT2-mCherry. At 2 dpt, cells were fixed and stained with DAPI. The boxed area is enlarged 4× on the right. Bar, 10 μm. (B) Colocalization of NPC1^P692S^-GFP or NPC1-GFP with FLOT2-mCherry was quantified by counting vesicles in 30 cells per group from three independent experiments. *, *P < *0.05, two-tailed *t* test. (C) HEK293T cells transfected with NPC1-GFP or NPC1^P692S^-GFP at 3 dpt were lysed and immunoprecipitated (IP) with anti-GFP affinity gel. Whole-cell lysates and immunoprecipitates were analyzed by Western blotting (WB) with antibodies against GFP, FLOT2, and tubulin. (D) Relative ratio of WB band intensities, with the ratio of FLOT2/NPC1-GFP set as 100%. Results are presented as the mean ± standard deviation from three independent experiments. *, *P < *0.05, two-tailed *t* test.

A single-amino-acid mutation of the sterol-sensing domain of NPC1, namely, NPC1^P692S^, results in decreased LDL-derived cholesterol delivery to endoplasmic reticulum and the plasma membrane, similar to what is observed in Niemann-Pick type C disease (20, 21). Given the requirement of the FLOT2 CRAC motifs for FLOT2 colocalization with NPC1 (Fig. 3A and B), we examined whether the NPC1 sterol-sensing domain is required for this colocalization. FLOT2-mCherry localized mainly to the plasma membrane in NPC1^P692S^-GFP-cotransfected cells (Fig. 4A); consequently, colocalization of FLOT2-mCherry with NPC1^P692S^-GFP vesicles was significantly reduced compared with the NPC1-GFP control (Fig. 4A and B). Indeed, the amount of endogenous FLOT2 that was pulled down with anti-GFP from the lysate of HEK293T cells transfected with NPC1^P692S^-GFP was significantly reduced compared with cells transfected with NPC1-GFP (Fig. 4C and D). Thus, the sterol-sensing domain of NPC1, which is critical for normal intracellular cholesterol distribution, is required for FLOT2-NPC1 colocalization and interaction.

### Cholesterol dependence of the colocalization/interaction of FLOT2 with NPC1.

**FIG 5:**
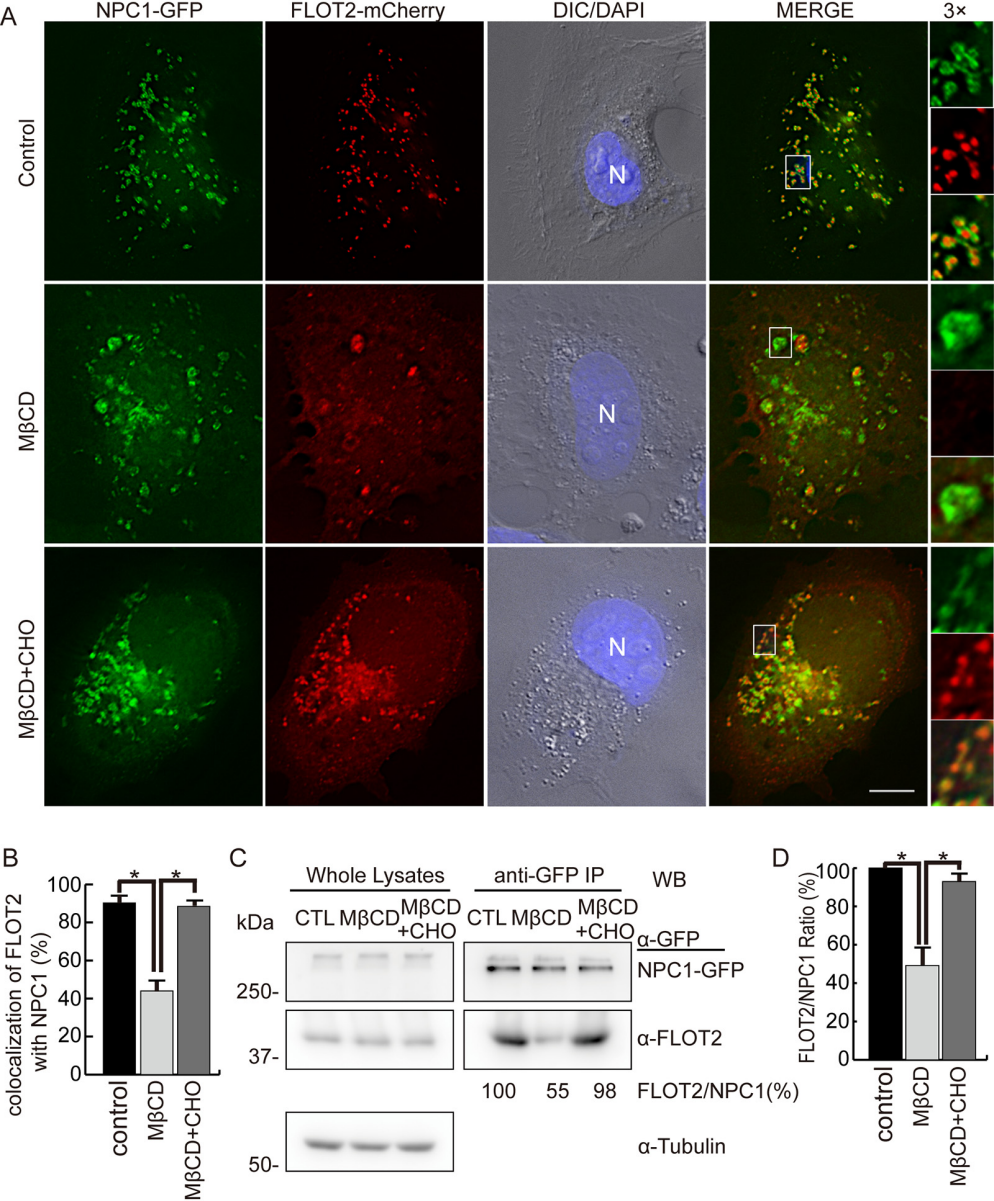
MβCD reduces colocalization as well as the physical interaction of NPC1 with FLOT2. (A) RF/6A cells were cotransfected with plasmids NPC1-GFP and FLOT2-mCherry, and at 2 dpt cells were fixed and stained with DAPI. Alternatively, cells were treated with 2.5 mM MβCD or MβCD supplemented with 20 μg/ml water-soluble cholesterol (MβCD+CHO) for 40 min prior to fixation. The boxed area is enlarged 3× on the right. Bar, 10 μm. (B) Colocalization between NPC1-GFP and FLOT2-mCherry was quantified by counting vesicles in 30 cells per group from three independent experiments. *, *P < *0.05 by ANOVA. (C) NPC1-GFP-transfected HEK293T cells at 3 dpt were not treated (control, CTL) or were treated with 2.5 mM MβCD or MβCD supplemented with 20 μg/ml water-soluble cholesterol (MβCD+CHO) for 40 min prior to harvesting. Lysates were immunoprecipitated (IP) with anti-GFP nanobody affinity gel. Whole-cell lysates and immunoprecipitates were analyzed by Western blotting (WB) with antibodies against GFP, FLOT2, and tubulin. (D) Relative ratio of WB band intensities, with the ratio of CTL set as 100%. Results are presented as the mean ± standard deviation from three independent experiments. *, *P < *0.05 by ANOVA.

FLOTs have two major subcellular localization pools, namely, the plasma membrane and intracellular vesicles, and this differential localization is regulated by free cholesterol (15, 17). An increase in cellular cholesterol above a certain threshold leads to transfer of FLOTs from the plasma membrane to intracellular vesicles, and, reciprocally, cholesterol depletion drives vesicular FLOT2 to the plasma membrane (17). Given that the CRAC domain of FLOT2 is required for FLOT2 colocalization with NPC1 (Fig. 3A and B) and the sterol-sensing domain of NPC1 is critical for FLOT2 and NPC1 colocalization and interaction (Fig. 4), we examined the requirement of cellular cholesterol for the FLOT2-NPC1 interaction using methyl-β-cyclodextrin (MβCD), which, at 10 mM concentration, reduces membrane cholesterol abundance by inducing cellular cholesterol efflux (22). As 10 mM MβCD caused RF/6A cells to retract and partially detach from the substratum, we treated RF/6A cells with 2.5 mM MβCD for 40 min at 2 days posttransfection (dpt). This treatment greatly reduced FLOT2 localization to intracellular vesicles; consequently, the colocalization between NPC1-GFP and FLOT2-mCherry was significantly decreased compared with the control (Fig. 5A and B). After cholesterol replenishment in MβCD-treated cells for 40 min, the colocalization was restored (Fig. 5A and B). Treatment with MβCD significantly reduced the amount of endogenous FLOT2 that was pulled down with anti-GFP from the lysate of HEK293T cells transfected with NPC1-GFP (Fig. 5C and D), and cholesterol replenishment restored the FLOT2 and NPC1-GFP interaction (Fig. 5C and D). Thus, colocalization and interaction of FLOT2 and NPC1 in the cell are dependent on membrane cholesterol level.

### Ezetimibe blocks the colocalization/interaction of FLOT2 with NPC1.

Ezetimibe, which blocks cholesterol absorption at the brush border of the small intestine, is an FDA-approved LDL cholesterol-lowering drug for the treatment of hypercholesterolemia (23, 24). Altmann et al. (25) reported the discovery of the Niemann-Pick C1-like 1 protein (NPC1L1) as the human sterol transport protein that was expressed at the enterocyte lumenal (apical) surface as well as the hepatobiliary (canalicular) interface. NPC1L1 was then identified as the molecular target of ezetimibe, and the ezetimibe-binding site of NPC1L1 was determined (26). FLOTs play a critical role in this NPC1L1-mediated cholesterol uptake via formation of cholesterol-enriched membrane microdomains, which function as carriers for the bulk of cholesterol (27). Ezetimibe binding to NPC1L1 disrupts the formation of the NPC1L1-FLOT1/2 complex, resulting in reduced cholesterol absorption via NPC1L1-mediated endocytosis (27).

**FIG 6:**
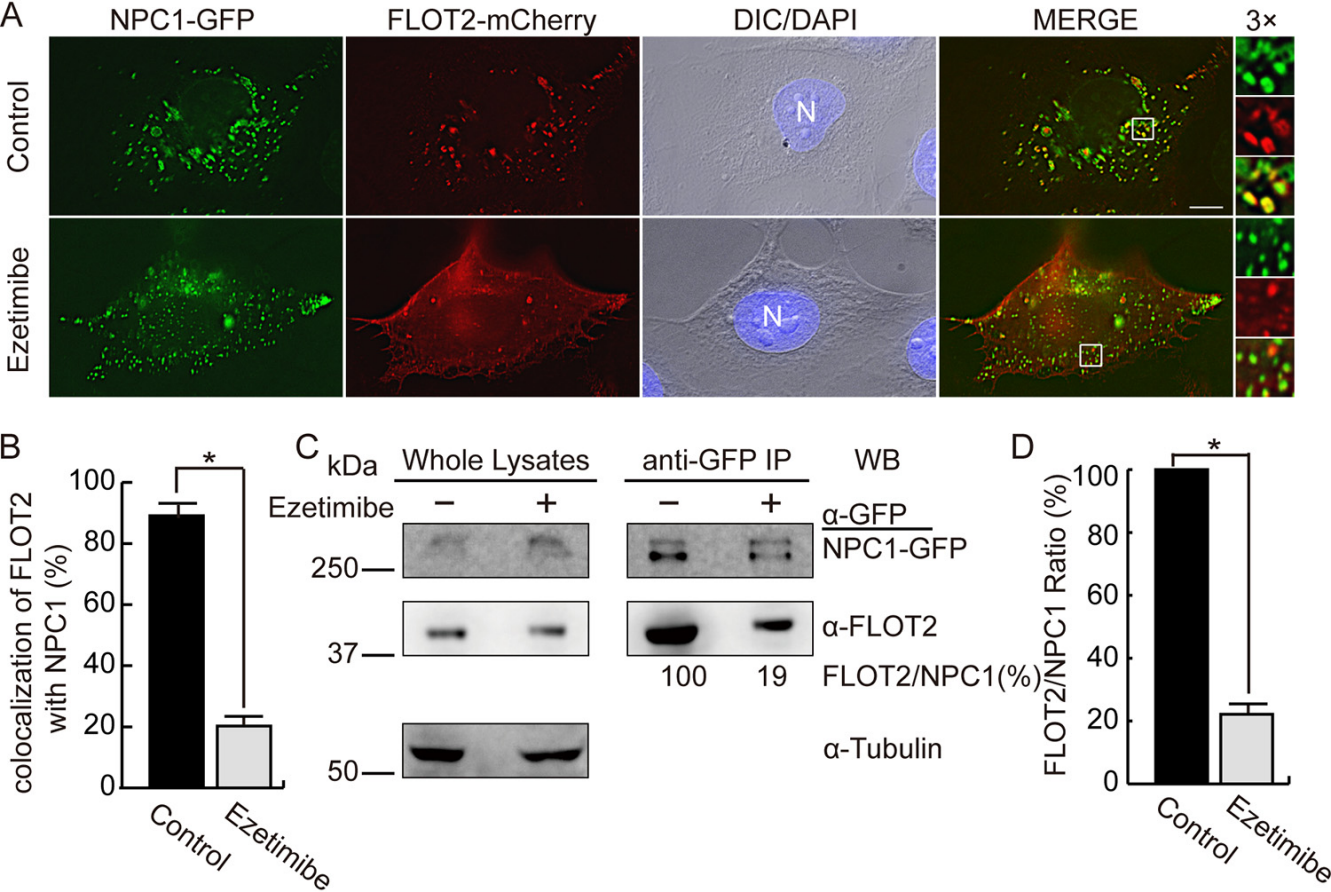
Ezetimibe reduces colocalization of NPC1 and FLOT2. (A) RF/6A cells were cotransfected with NPC1-GFP and FLOT2-mCherry. At 2 dpt, cells were treated with DMSO control or 40 μM ezetimibe for 20 h and then fixed and stained with DAPI. The boxed area is enlarged 3× on the right. Bar, 10 μm. (B) Quantification of colocalization of NPC1-GFP with FLOT2-mCherry, with 30 cells counted per group from three independent experiments. *, *P < *0.05 by ANOVA. (C) Coimmunoprecipitation of NPC1-GFP and endogenous FLOT2. NPC1-GFP-transfected HEK293T cells at 2 dpt were treated with DMSO (control) or 40 μM ezetimibe for 20 h. Lysates were immunoprecipitated (IP) with anti-GFP affinity gel. Whole-cell lysates and immunoprecipitates were analyzed by Western blotting (WB) with antibodies against GFP, FLOT2, and tubulin. (D) Relative ratio of WB band intensities, with the ratio of untreated (control) set as 100%. Results are presented as the mean ± standard deviation from three independent experiments. *, *P < *0.05 by two-tailed *t* test.

Based on NCBI conserved domain structure analysis, human NPC1 and NPC1L1 proteins have similar Niemann-Pick C type protein family domains, which include an NPC1 N-terminus domain (pfam16414) with a cholesterol-binding pocket and a sterol-sensing domain (pfam12349) at the central region (see Fig. S1A in the supplemental material). In addition, alignment showed that these two proteins share 40% amino acid identity and 57% amino acid similarity (Fig. S1B and C). Thus, we tested the effects of ezetimibe on the FLOT2-NPC1 interaction. When RF/6A cells were cotransfected with FLOT2-mCherry and NPC1-GFP and treated with 40 μM ezetimibe for 20 h at 2 dpt, FLOT2-mCherry localized mainly to the plasma membrane; consequently, colocalization between FLOT2-mCherry and NPC1-GFP was significantly reduced (Fig. 6A and B). Treatment with ezetimibe significantly reduced the amount of endogenous FLOT2 that was pulled down with anti-GFP from the lysate of HEK293T cells transfected with NPC1-GFP (Fig. 6C and D).

10.1128/mBio.02299-21.1 FIG S1 Human NPC1 and NPC1L1 share similar domain structures. (A) NCBI conserved domain search showed that human NPC1 and NPC1L1 proteins ((NCBI accession numbers NP_000262.2 and NP_037521.2, respectively) have similar Niemann-Pick C type protein family domains, which include an NPC1 N-terminus domain (NPC1_N, pfam16414) with a cholesterol-binding pocket and a sterol-sensing domain (pfam12349) at the central region. (B and C) Human NPC1 and NPC1L1 proteins were aligned using Clustal Omega with BLOSUM62 matrix. Alignment of human NPC1 and NPC1L1 proteins showed that they share 40% amino acid identity, or 57% similarity. Download FIG S1, TIF file, 2.7 MB. Copyright © 2021 Huang et al. 2021 Huang et al. https://creativecommons.org/licenses/by/4.0/ This content is distributed under the terms of the Creative Commons Attribution 4.0 International license.

### Endogenous FLOT2, endogenous NPC1, and HA-FLOT2^1–183^ traffic to the lumen of A. phagocytophilum inclusions and encase individual bacteria.

**FIG 7:**
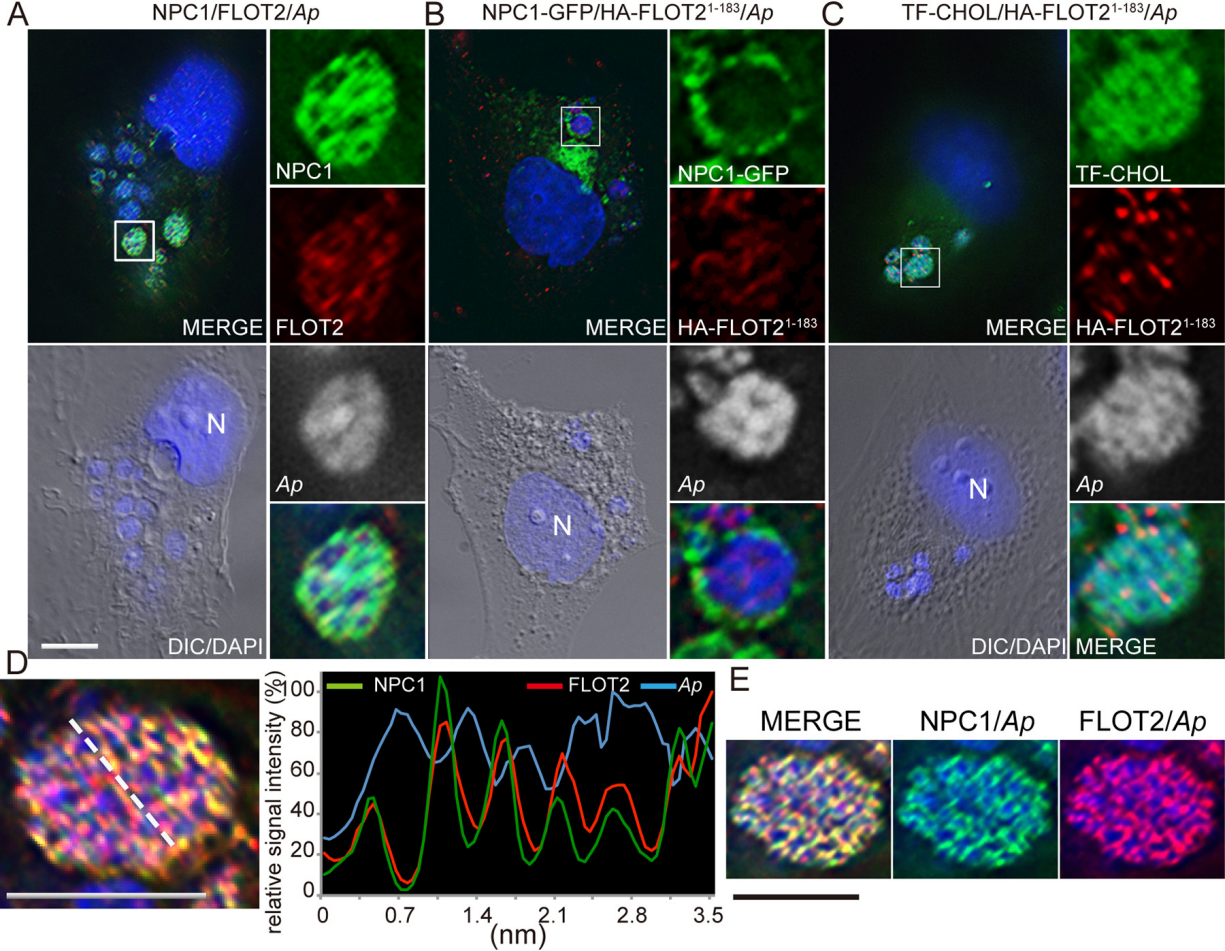
FLOT2^1–183^ localizes inside A. phagocytophilum inclusions. (A) A. phagocytophilum (*Ap*)-infected RF/6A cells at 3 dpi were fixed and dually labeled with rabbit anti-NPC1 and mouse anti-FLOT2. (B and C) RF/6A cells were transfected with NPC1-GFP and HA-FLOT2^1–183^ (B) or HA-FLOT2^1–183^ (C). At 4 hpt, cells were infected with A. phagocytophilum for 3 days (B and C) and then incubated with 2 μM TF-cholesterol in AMEM containing 5% LPDS (C). Cells were fixed and labeled with anti-NPC1 and/or anti-HA. (A to C) Cells were treated with the serine protease inhibitor DFP before fixation. A. phagocytophilum and host cell nuclei were labeled by DAPI (blue colored in the merged channels, gray pseudocolored in the enlarged panels). The boxed area is enlarged 4× on the right. Bar, 10 μm. (D) Relative signal intensity profiles of green (NPC1), red (FLOT2), and blue (DAPI, A. phagocytophilum DNA) fluorescence along the white dashed line from an inclusion in other cells labeled as described for panel A, after normalizing to the highest fluorescence intensity. (E) 3D-rendered image of panel D constructed with ImageJ by stacking 11 layers of 0.1-μm thickness of DeltaVision images. (D and E) Bar, 5 μm.

Given that each of FLOT2 and NPC1 localizes to A. phagocytophilum inclusions (6, 16, 28) and the interaction of FLOT2 and NPC1 in uninfected cells (Fig. 1 to 6), we examined whether they colocalize on A. phagocytophilum inclusions. Immunofluorescence labeling showed endogenous FLOT2 and NPC1 were colocalized within A. phagocytophilum inclusions, encasing individual bacteria (Fig. 7A). Indeed, the fluorescence intensity profile of red (FLOT2), green (NPC1), and blue (4′,6′-diamidino-2-phenylindole [DAPI]; A. phagocytophilum DNA) signals along the length of the line of an A. phagocytophilum inclusion revealed that the multipeak red signals overlap green signals and were reciprocated with blue signals (Fig. 7D). The three-dimensional (3D) shadow projection image, constructed based on the Z-stack data from DeltaVision microscopy, affirmed FLOT2 and NPC1 colocalization within A. phagocytophilum inclusions (Fig. 7E).

As FLOT2^1–183^ was sufficient for localization within NPC1-GFP vesicles (Fig. 2A and B), we next examined FLOT2^1–183^ localization to A. phagocytophilum inclusions. As the mCherry tag was too bulky to effectively track FLOT2^1–183^, hemagglutinin (HA)-tagged FLOT2^1–183^ was constructed. HA-FLOT2^1–183^ clearly localized within inclusions, whereas NPC1-GFP, unlike endogenous NPC1, remained on the inclusion membrane (Fig. 7B), as previously reported (6). HA-FLOT2^1–183^ encased each of the DAPI-stained bacteria within the inclusions (Fig. 7B).

We previously showed that the lumen of A. phagocytophilum inclusions is enriched with cholesterol based on staining with filipin, a free cholesterol-binding polyene antibiotic (5). Dipyrromethene difluoride-cholesterol (BODIPY- or TopFluor-cholesterol [TF-cholesterol]) is a widely used cholesterol analog because it has greater intrinsic fluorescence (bright and photostable) than filipin and partitions in membranes similarly to natural cholesterol (29, 30). When dissolved in solvent and applied to cells in growth medium containing lipoprotein-deficient serum, TF-cholesterol diffuses into eukaryotic cells and equilibrates slowly with intracellular membranes (30). We previously used this approach to more clearly visualize the distribution of host membrane cholesterol in inclusions of another cholesterol-dependent bacterium, Ehrlichia chaffeensis (31). In agreement with our previous study with filipin (5) and others (28), TF-cholesterol was highly enriched in A. phagocytophilum inclusion and colocalized with HA-FLOT2^1–183^ within the inclusions (Fig. 7C).

### NPC1^P692S^ cannot localize to A. phagocytophilum inclusions, and its overexpression reduces A. phagocytophilum infection.

**FIG 8:**
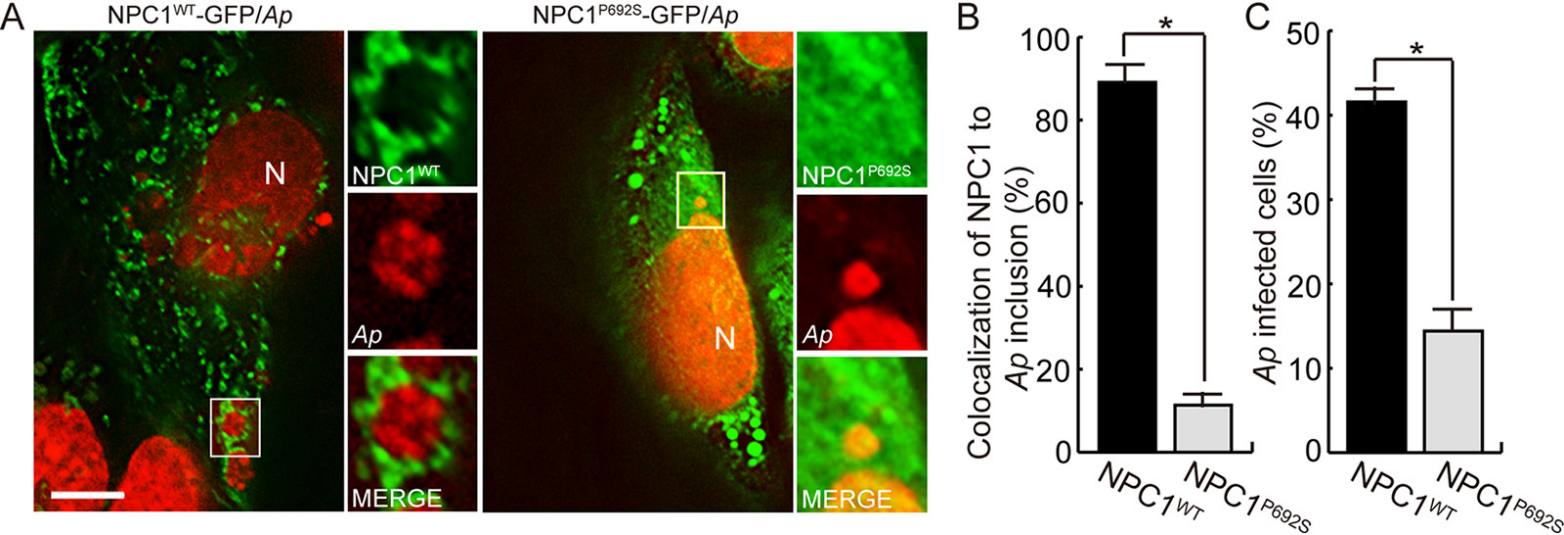
NPC1^P692S^-GFP does not localize to A. phagocytophilum inclusions and reduces A. phagocytophilum infection. (A) A. phagocytophilum*-*infected RF/6A cells at 1 dpi were transfected with wild-type NPC1 (NPC1^WT^)-GFP or NPC1^P692S^-GFP. Cells were fixed and stained with DAPI (pseudocolored in red) at 2 dpt/3 dpi. The boxed area is enlarged 3× on the right. Bar, 10 μm. (B) Quantification of colocalization of NPC1-GFP or NPC1^P692S^-GFP with A. phagocytophilum inclusions, with 200 inclusions counted per group from three independent experiments. (C) Percentage of NPC1^WT^- or NPC1^P692S^-GFP-expressing RF/6A cells infected with A. phagocytophilum, with 100 cells counted each from three independent experiments (mean ± standard deviation). *, *P < *0.05 by two-tailed *t* test.

As NPC1^P692S^ had reduced colocalization or interaction with FLOT2 (Fig. 4A to D), we examined whether NPC1^P692S^ could localize to A. phagocytophilum inclusions. Indeed, compared to NPC-1-GFP, NPC1^P692S^-GFP localization to inclusions (Fig. 8A and B) and the number of infected cells was significantly reduced (Fig. 8C), indicating that the sterol-sensing domain of NPC1 is required for both localization of NPC1 to inclusions and delivery of cholesterol to A. phagocytophilum to support its proliferation.

### Ezetimibe blocks A. phagocytophilum infection in HL-60 and RF/6A cells.

**FIG 9:**
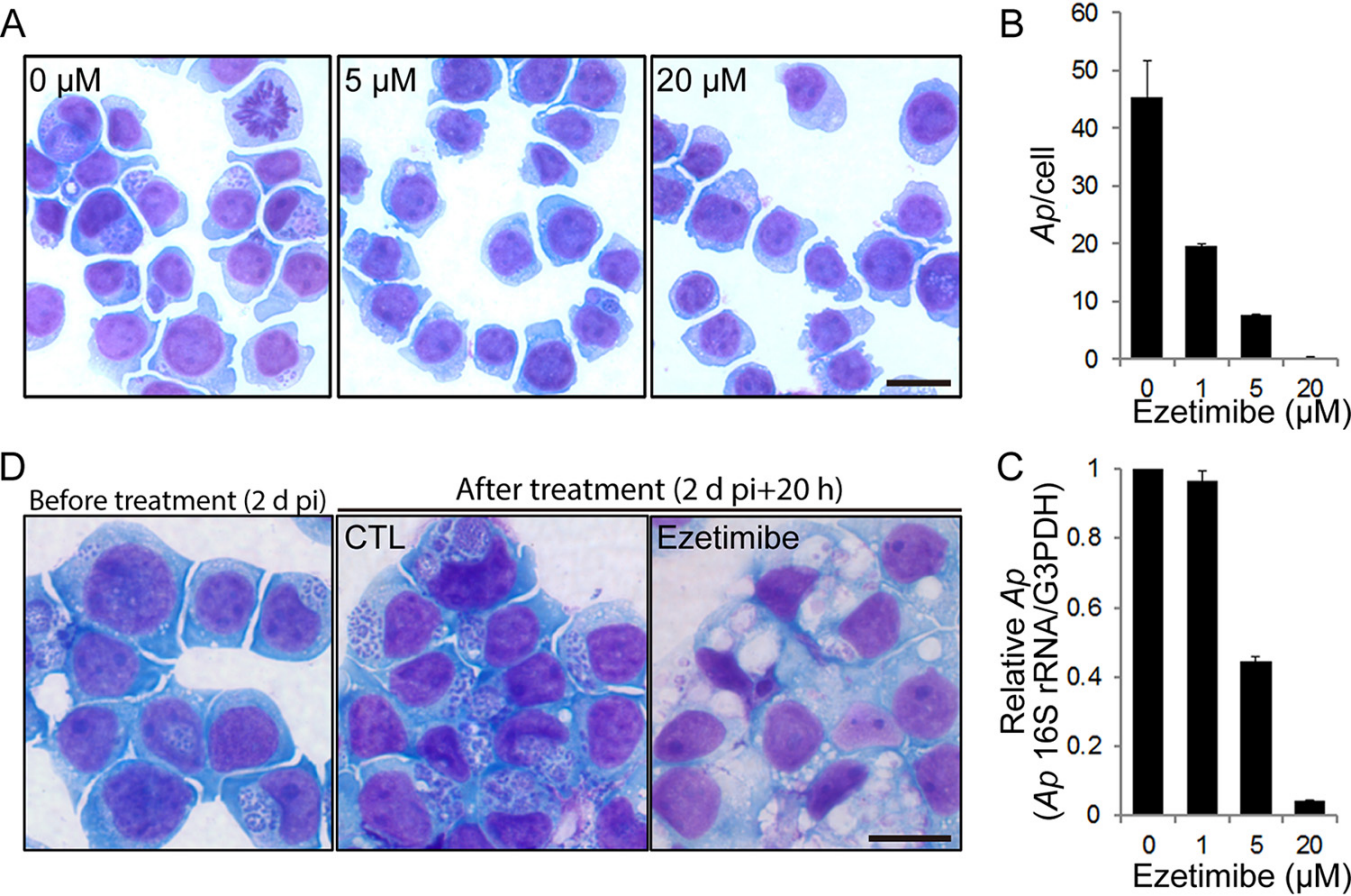
Ezetimibe inhibits A. phagocytophilum infection in HL-60 cells. (A to C) A. phagocytophilum (*Ap*)-infected HL-60 cells at 2 hpi were treated with ezetimibe (1, 5, or 20 μM) for 2 days. Bar, 10 μm. (B) The number of A. phagocytophilum cells was scored in 200 host cells in triplicate culture wells at 2 dpi by Diff-Quik staining. (C) Quantitative PCR for detecting A. phagocytophilum 16S rRNA gene normalized by human *G3PDH.* (B and C) Results are presented as the mean ± standard deviation from three independent experiments. *, *P* < 0.05 by ANOVA. (D) A. phagocytophilum*-*infected HL-60 cells at 2 dpi were treated with DMSO control (CTL) or ezetimibe (40 μM) for 20 h. Diff-Quik staining was used. Bar, 10 μm.

**FIG 10:**
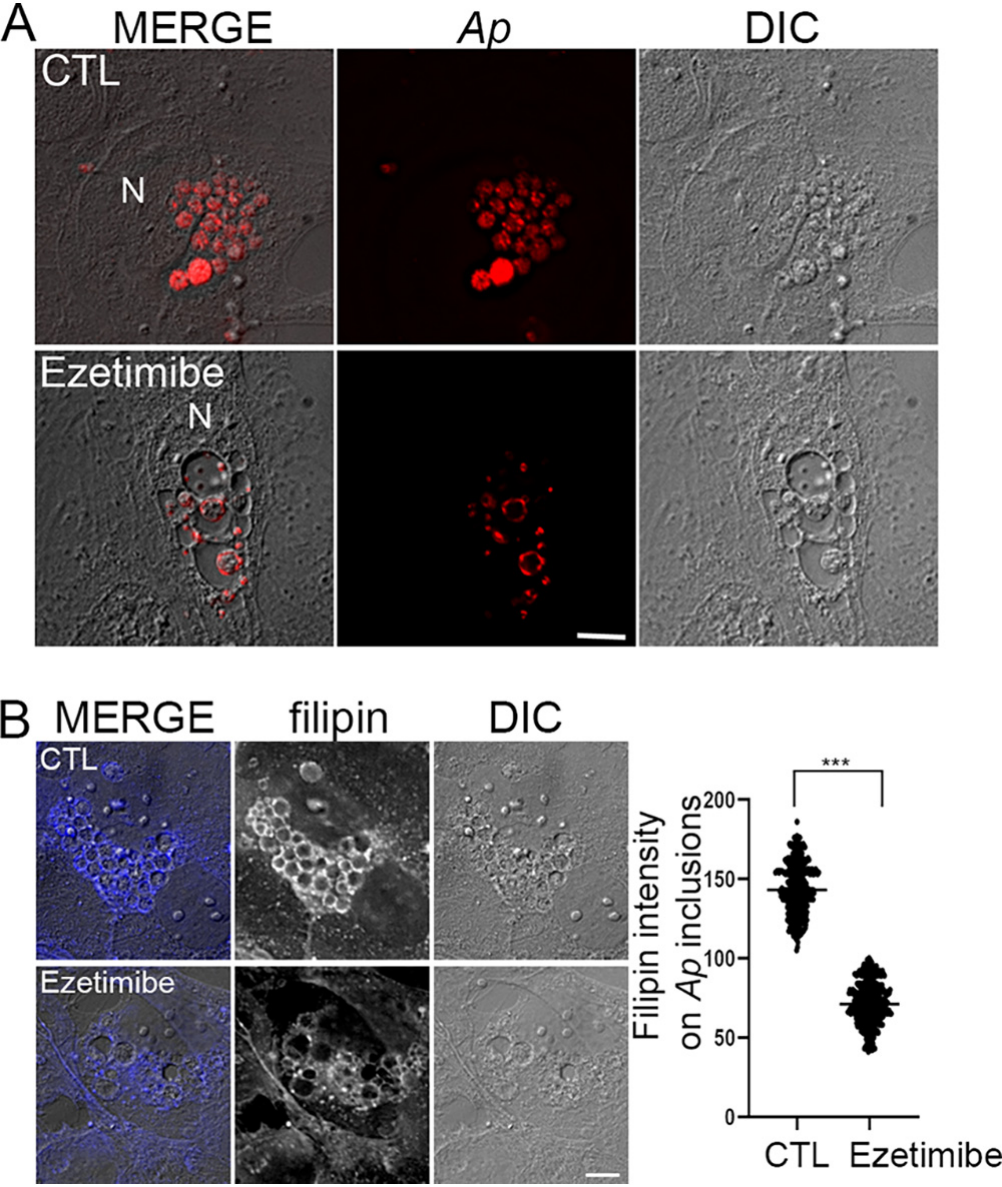
Ezetimibe enlarges A. phagocytophilum vacuole size and reduces cholesterol accumulation in A. phagocytophilum vacuoles. (A) A. phagocytophilum (*Ap*)-infected RF/6A cells at 1.5 dpi were treated with DMSO control (CTL) or ezetimibe (20 μM) for 1 day. The cells were fixed and immunostained for A. phagocytophilum outer membrane protein P44. N, nucleus. Merged/DIC, image merged with differential interference contrast. Bar, 5 μm. The results are representative of at least four independent experiments. (B) A. phagocytophilum*-*infected RF/6A cells at 1 dpi were treated with DMSO control (CTL) or ezetimibe (10 μM) for 2 days. The cells were fixed and labeled with filipin (pseudocolored in gray in the individual channels). The intensities of the filipin signal in individual inclusions in ezetimibe-treated (*n* = 30) and control (*n* = 30) cells were measured using ImageJ. ***, *P < *0.001, two-tailed *t* test.

Because NPC1 and FLOT2 are required for A. phagocytophilum infection (6, 16) and we found that ezetimibe blocks NPC1-FLOT2 colocalization and interaction (Fig. 6), we examined whether ezetimibe could block infection of cells with A. phagocytophilum. First, we demonstrated that ezetimibe at 2 to 40 μM was not toxic to HL-60 cells (Fig. S2). Ezetimibe then was added to HL-60 cells in culture at 2 h postinfection (hpi; just after internalization and when bacterial inclusions were not discernible in the cells under a light microscope). Cells were then harvested at 2 days postinfection (dpi). Ezetimibe indeed blocked A. phagocytophilum proliferation in a dose-dependent manner (1 to 20 μM; Fig. 9A to C). In addition, the ezetimibe-mediated inhibition of proliferation could be achieved even when ezetimibe was added to the cell culture at 2 dpi with subsequent incubation for only 20 h (Fig. 9D). Strikingly, with ezetimibe treatment, many vacuoles containing lightly stained materials and a few bacteria, were seen in the cytoplasm of the infected HL-60 cells, in contrast to the presence of numerous A. phagocytophilum inclusions tightly packed with numerous bacteria in nontreated control cells (Fig. 9D). Notably, ezetimibe did not directly inhibit A. phagocytophilum, as demonstrated by the fact that infection was not blocked when host cell-free A. phagocytophilum was treated with ezetimibe (10 μM) for 30 min and then added to HL-60 cells after removal of ezetimibe (Fig. S3). The inhibition of A. phagocytophilum infection and vacuolation caused by ezetimibe were not specific to the host cell type, as similar results were obtained with RF/6A cells (Fig. S4). The vacuolation was observed specifically when A. phagocytophilum*-*infected cells were treated 1 to 2 dpi with ezetimibe, as 0% of cells developed vacuolation when uninfected cells were treated (Fig. S4F), or infected cells were treated starting 2 hpi with ezetimibe (Fig. 9). Ezetimibe-induced inhibition of A. phagocytophilum proliferation and vacuolation were reversible, as demonstrated by the fact that when ezetimibe was added at 24 hpi and removed at 36 hpi, regrowth and reduction of vacuoles were observed at 72 hpi (Fig. S5). Vacuolization was confirmed as being a consequence of ezetimibe-mediated modifications of A. phagocytophilum inclusions, as intravacuolar A. phagocytophilum could be detected by immunofluorescence staining with monoclonal antibody 5C11, which is specific for the A. phagocytophilum outer membrane protein P44 (32) (Fig. 10A). Lastly, we used filipin staining to examine whether free cholesterol levels associated with A. phagocytophilum inclusions were altered with/without ezetimibe treatment. Indeed, the level of inclusion-associated free cholesterol was significantly lower in ezetimibe-treated cells (Fig. 10B). Taken together, these data indicated that ezetimibe could block A. phagocytophilum infection in host cells by preventing (or even reversing) cholesterol trafficking to A. phagocytophilum inclusions.

10.1128/mBio.02299-21.2 FIG S2 Ezetimibe has very low toxicity for HL-60 cells. Ezetimibe was added to HL-60 cells at the indicated concentrations (0 to 40 μM). Cell density at 1 and 2 days posttreatment was determined by counting cells (A), and cell viability was determined with the trypan blue exclusion assay (B). Results are presented as the mean ± standard deviation from three independent experiments. ns, no significant difference by ANOVA. Download FIG S2, TIF file, 0.2 MB. Copyright © 2021 Huang et al. 2021 Huang et al. https://creativecommons.org/licenses/by/4.0/ This content is distributed under the terms of the Creative Commons Attribution 4.0 International license.

10.1128/mBio.02299-21.3 FIG S3 Pretreatment of A. phagocytophilum does reduce bacterial infection of host cells. Host cell-free A. phagocytophilum (*Ap*) was treated with ezetimibe (10 μM) for 30 min at 37°C and then added into HL-60 cells after washing out ezetimibe. Bacterial infection was scored at 2 dpi by quantitative PCR for detection of A. phagocytophilum 16S rRNA. Human *G3PDH* was used to normalize the data. Results are presented as the mean ± standard deviation from three independent experiments. ns, no significant difference by two-tailed *t* test. Download FIG S3, TIF file, 0.2 MB. Copyright © 2021 Huang et al. 2021 Huang et al. https://creativecommons.org/licenses/by/4.0/ This content is distributed under the terms of the Creative Commons Attribution 4.0 International license.

10.1128/mBio.02299-21.4 FIG S4 Ezetimibe inhibits A. phagocytophilum infection in RF/6A cells and causes vacuolation of A. phagocytophilum inclusions in infected host cells. (A and B) Ezetimibe (20 μM) was added into A. phagocytophilum*-*infected RF/6A cells at 2 hpi and kept in the medium for 2 days. The percentage of infected cells was determined at 2 dpi by Diff-Quik staining. *Ap*, A. phagocytophilum. Results are presented as the mean ± standard deviation from three independent experiments. *, *P < *0.05 by ANOVA. (C) Ezetimibe (20 μM) was added into A. phagocytophilum*-*infected RF/6A cells at 2 dpi and kept in the medium for 1 day. Diff-Quik staining was used. (D to F) Ezetimibe (20 μM) was added to A. phagocytophilum*-*infected RF/6A cells during 1 to 2 dpi at different time points with subsequent incubation for 4, 8, or 24 h. The cells were checked at 2 dpi, and typical morphological changes after treatment were determined by Diff-Quik staining. (E) Percentage of vacuolated A. phagocytophilum inclusions in infected RF/6A cells at different treatment times (4, 8, or 24 h). (F) No vacuole was observed in uninfected RF/6A cells after 24 h of treatment with ezetimibe. Download FIG S4, TIF file, 2.9 MB. Copyright © 2021 Huang et al. 2021 Huang et al. https://creativecommons.org/licenses/by/4.0/ This content is distributed under the terms of the Creative Commons Attribution 4.0 International license.

10.1128/mBio.02299-21.5 FIG S5 Inhibition of ezetimibe on A. phagocytophilum infection is reversible. Ezetimibe (EZ; 10 μM) was added to A. phagocytophilum*-*infected HL-60 at 24 hpi and was washed out of the culture medium at 36 or 48 hpi. Infected cells were maintained in culture in fresh medium for 24 h, and the infection was scored at 72 hpi by Diff-Quik staining (A) and by quantitative PCR (B). Results are presented as the mean ± standard deviation from three independent experiments. *, *P < *0.05 by ANOVA. Download FIG S5, TIF file, 1.4 MB. Copyright © 2021 Huang et al. 2021 Huang et al. https://creativecommons.org/licenses/by/4.0/ This content is distributed under the terms of the Creative Commons Attribution 4.0 International license.

### Ezetimibe blocks trafficking of host cell membrane lipids to A. phagocytophilum inclusions.

**FIG 11:**
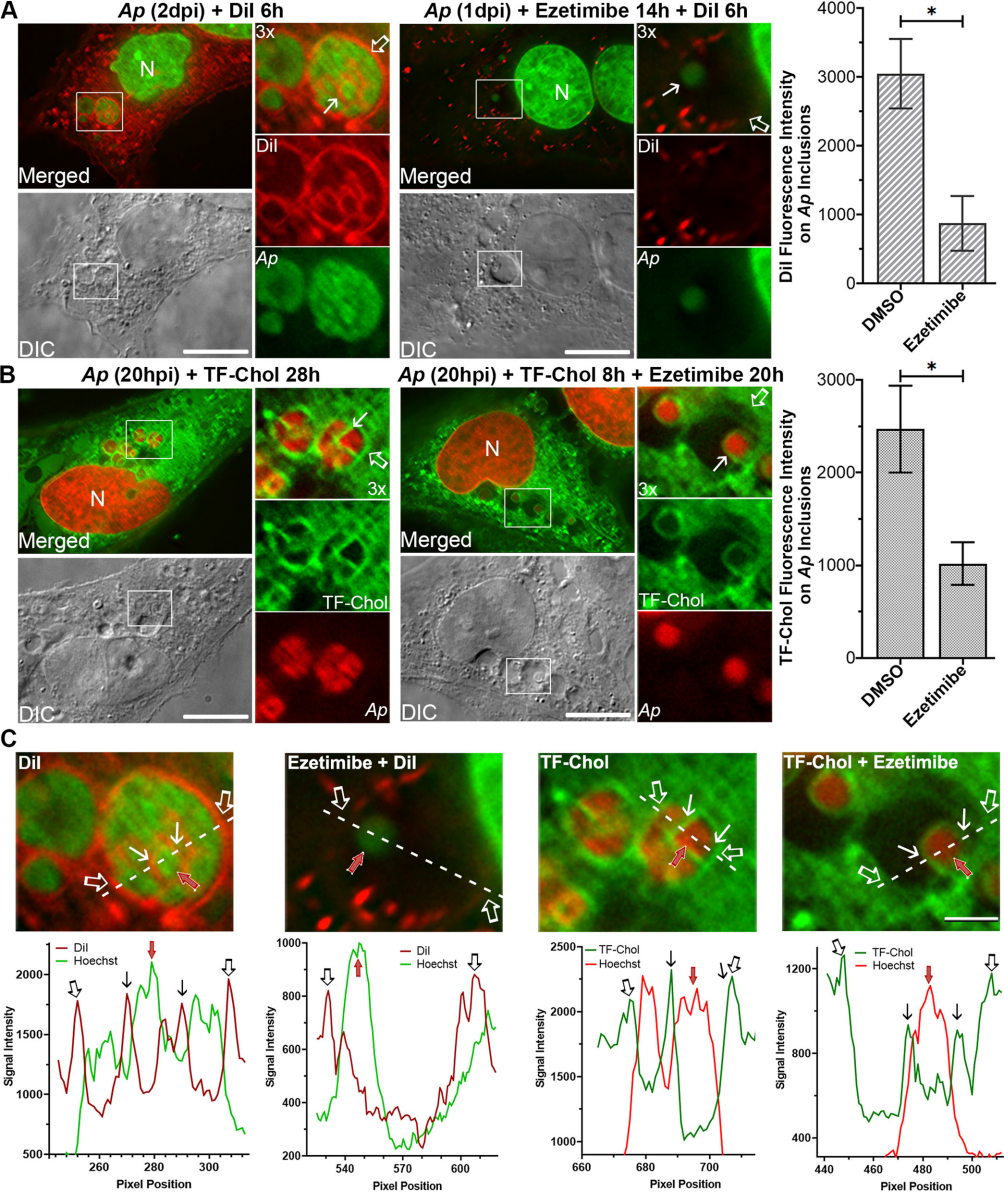
Ezetimibe blocks trafficking of host-cell membrane lipids to A. phagocytophilum inclusions. (A) A. phagocytophilum (*Ap*)-infected RF/6A cells at 1 dpi were treated with DMSO control or 40 μM ezetimibe for 14 h and then incubated with 5 μM DiI for 6 h in the presence of ezetimibe. (B) Alternatively, at 20 hpi, cells were incubated with 2 μM TF-cholesterol in AMEM containing 5% LPDS for 8 h and then treated with DMSO control or 40 μM ezetimibe for 20 h in the presence of TF-cholesterol. Cells were fixed and DNA was stained by Hoechst 33342. A DeltaVision microscope was used. Hoechst 33342 labeling was pseudocolored in green (A) or red (B) for better visualization, and boxed areas in the merged images were enlarged 3× at the right. DIC, differential interference contrast; open arrows, inclusion membranes; solid arrows, A. phagocytophilum membranes; N, nucleus. Bar, 10 μm. Images are representative of three independent experiments. Fluorescence intensities of individual inclusion membranes were measured using Image J by counting at least 10 cells per group for each experiment, and results were shown as mean ± standard deviation. *, significantly different by Student’s unpaired *t* test (*P < *0.01). (C) Line-profile analysis of fluorescence intensity signals on A. phagocytophilum inclusions along the dotted white lines (boxed areas in panels A and B), which were labeled with DiI (A) or TF-Chol (B), and Hoechst 33342. Open arrows, inclusion membrane; solid arrows, A. phagocytophilum membrane; red arrows, A. phagocytophilum. Bar, 2 μm.

Using DiI [3,3′-dioctadecylindocarbocyanine, DiIC_18_(3)] to label host cell membranes (31, 33, 34), and using TF-cholesterol to visualize the distribution of membrane cholesterol (31), we examined whether ezetimibe affects the trafficking of host-derived membrane lipids to bacterium-replicating inclusions and A. phagocytophilum. To prevent diffusion of DiI between membranes, cells were fixed with paraformaldehyde (PFA) without permeabilization, and a coverslip sealant containing no organic solvents was used. Fluorescence microscopy and line profile analysis showed that DiI strongly labeled membranes of all A. phagocytophilum inclusions (Fig. 11A and C, open arrows) and most individual bacterium (Fig. 11A and C, solid arrows) in infected RF/6A cells, suggesting that host cell membranes were trafficked and incorporated into membranes of A. phagocytophilum inclusions and individual bacteria. Similarly, membranes of A. phagocytophilum inclusions and individual bacteria were extensively labeled by TF-cholesterol (Fig. 11B and C, open and solid arrows, respectively).

When A. phagocytophilum-infected RF/6A cells at 1 dpi were treated with ezetimibe for 20 h, bacterial growth and inclusion numbers were severely reduced, similar to results shown in Fig. 9 and Fig. S4, and trafficking of host membranes labeled by DiI- or TF-cholesterol to A. phagocytophilum inclusions were significantly reduced more than 2-fold (Fig. 11A and B). Interestingly, when A. phagocytophilum-infected cells were treated with ezetimibe for 14 h and then labeled with DiI for 6 h in the presence of ezetimibe, DiI labeling of A. phagocytophilum bacterial membrane within the inclusions was completely prevented (Fig. 11A and C). On the other hand, when TF-cholesterol was incubated with infected cells at 20 hpi for 8 h and then treated with ezetimibe for 20 h, a small number of surviving bacteria retained TF-cholesterol labeling (Fig. 11B and C). Taken together, these data suggest that A. phagocytophilum in the inclusions incorporates host membrane lipids and cholesterol from the host membrane vesicles, which can be blocked by ezetimibe.

## DISCUSSION

A. phagocytophilum captures cholesterol exclusively from LDL (5), and NPC1 and FLOT2 both target A. phagocytophilum inclusions and are required for infection (6, 16). However, the relationship between NPC1 and FLOTs was unknown in uninfected cells, not to mention infected cells. One of the important findings of the present study is the striking presence of FLOT2 in the NPC1-containing vesicles and their physical interaction. We previously showed that FLOT2 localizes on LDL- and acid lipase-containing vesicles (16). Collectively, our results revealed the critical role of FLOT in the normal physiological process of intracellular membrane cholesterol trafficking, including LDL-derived cholesterol from acidic endosomes to the NPC1 compartment, which is the main sorting compartment for cellular cholesterol.

FLOT2-GFP bearing two mutations in the CRAC motif cannot target to A. phagocytophilum inclusions (16). The cholesterol-sequestering agent MβCD abrogated FLOT2 localization to A. phagocytophilum inclusions and cleared infection (16). The present study revealed the FLOT2 CRAC mutant could not interact with NPC1 vesicles, and MβCD reduced FLOT2 interaction with NPC1. The NPC1 sterol-sensing domain mutant NPC1^P692S^ sequesters cholesterol within phagolysosomes and lysosomes rather than redistributing it to the plasma membrane and multivesicular bodies (20, 21); consequently, FLOT2 did not interact with NPC1^P692S^, and NPC1^P692S^ could not target A. phagocytophilum inclusions. Similar to the effects of U18666A treatment, which sequesters cholesterol within phagolysosomes (6), overexpression of NPC1^P692S^ also reduced A. phagocytophilum infection. Taken together, the present study illuminated that the cholesterol-dependent NPC1 and FLOT2 interaction is critical for A. phagocytophilum to hijack the intracellular LDL-cholesterol transport process to capture cholesterol for proliferation.

The presence of multitransmembrane cholesterol-binding protein NPC1, lipid raft-binding protein FLOT2, or lipid raft-binding FLOT2 SPFH domain within A. phagocytophilum inclusions, encasing individual bacteria, suggests there are cholesterol-rich intraluminal membranes in addition to membranes of A. phagocytophilum. We recently reported that intrainclusion membranes are abundant in vacuoles containing another cholesterol-dependent bacterium, Ehrlichia chaffeensis (31). Labeling of host cell membrane lipids, glycerophospholipids, and cholesterol, revealed that host cell membrane lipids are delivered into the lumen of *E. chaffeensis* inclusions, and this process is driven by bacterial factors (31). The present study revealed host membrane lipids and cholesterol are similarly transported into A. phagocytophilum inclusions, encasing individual bacteria. As NPC1 trafficking to A. phagocytophilum inclusions requires bacterial protein synthesis (6), A. phagocytophilum actively utilizes the NPC1-FLOT2-coordinated intracellular cholesterol transport mechanism to divert membrane cholesterol as well as membrane lipids into inclusions for bacterial proliferation.

Recently, NPC1 was shown to consist of two forms that localize to different organelles: the mannose-rich form (NPC1h), which is sensitive to endo H treatment (and, thus, is cleaved to a smaller polypeptide of ∼130 kDa), is located in the endoplasmic reticulum, and another form is the endo H-resistant complex glycosylated form (NPC1c) of ∼190 kDa, which is processed from NPC1h in the Golgi and ultimately traffics to lysosomes (35). The investigators showed that NPC1c is the exclusive form of NPC1 that fractionates in lipid rafts containing FLOT2. Although that study did not address the potential colocalization or physical interaction of NPC1 and FLOT2, their results suggest that the NPC1 physically interacting with FLOT2-containing vesicles in our study is the Golgi-processed NPC1c. The relative abundance of highly glycosylated NPC1 observed in A. phagocytophilum*-*infected cells (6) corroborates that the NPC1c- and FLOT2-containing lipid raft membrane is trafficked to A. phagocytophilum inclusions.

Another important finding of the present study is that the topological and physical association of NPC1 with FLOT2, and A. phagocytophilum proliferation can be blocked by ezetimibe. Unlike uninfected cells, treatment of infected cells with ezetimibe resulted in multiple intracellular vacuoles that were derived from former A. phagocytophilum inclusions. Similar phenomena were observed when A. phagocytophilum*-*infected cells were treated with oxytetracycline (6), which inhibits bacterial translation, or when *E. chaffeensis*-infected cells were treated with 3-methyl adenine, an autophagy inhibitor that prevents bacterial proliferation (36). In all these experimental scenarios, lysosomal fusion with bacterium-containing vacuoles was not observed, suggesting the bacteria could not maintain the inclusion integrity and starved to death owing to the inability to take up sufficient amounts of critical host cell factors, e.g., cholesterol, membrane lipids, and/or amino acids (6, 36). Ezetimibe inhibition of A. phagocytophilum proliferation and induction of vacuolation in infected cells were reversible. It seems once these cholesterol-dependent bacteria stop replicating, the intraluminal membranes, including the bacteria membrane, are integrated back to inclusions, which creates an enlarged vacuole.

Hydrophobic amines such as U18886A and imipramine are known to accumulate in acidic cellular compartments, particularly lysosomes, and block the postlysosomal transport of cholesterol in the LDL uptake pathway. We previously showed U18886A and imipramine significantly inhibit A. phagocytophilum infection and replication in HL-60 cells in a dose-dependent manner (5). Recently, it was reported that desipramine, a metabolite of imipramine and an acid sphingomyelinase inhibitor, blocks LDL-derived cholesterol efflux, similar to U18886A or imipramine, and significantly inhibits A. phagocytophilum infection in cell culture and in mice (28, 37). Taken together, these studies extend previous findings on the critical role of LDL-derived cholesterol for A. phagocytophilum proliferation (5, 6, 16); thus, LDL-derived cholesterol traffic can be a potential target of host-directed anti-A. phagocytophilum chemotherapy.

## MATERIALS AND METHODS

### Antibodies and plasmids.

The following antibodies were used: mouse monoclonal anti-FLOT2 (BD Pharmingen, San Jose, CA), rabbit anti-NPC1 (Novus Biologicals, Centennial, CO), mouse monoclonal anti-GFP (Santa Cruz Biotechnology, Santa Cruz, CA), mouse monoclonal anti-α-tubulin (Cell Signaling, Danvers, MA), and mouse monoclonal anti-hemagglutinin (HA) (Santa Cruz Biotechnology). Normal mouse IgG was purchased from Santa Cruz Biotechnology. Secondary antibodies conjugated to fluorescent probes (Alexa Fluor 488-conjugated goat anti-mouse IgG and Alexa Fluor 555-conjugated goat anti-mouse IgG) were obtained from Life Technologies (Eugene, OR), and peroxidase-conjugated secondary antibodies were obtained from KPL (Gaithersburg, MD).

The plasmid encoding NPC1-GFP was a gift from Matthew Scott (Addgene plasmid number 53521) (38). The plasmid encoding FLOT2-GFP was a gift from Verena Niggli from University of Bern, Switzerland (39). FLOT2-mCherry was amplified from the FLOT2-GFP plasmid and recloned into the pmCherry-N1 vector (Clontech, Mountain View, CA) at the XhoI and BamHI restriction sites (see Table S1 in the supplemental material). FLOT2^1–183^-GFP, FLOT2^1–183^-mCherry, and FLOT2^1–183^-HA were recloned from their respective full-length FLOT2 plasmids at the same restriction sites or by replacing the mCherry tag with an HA tag by PCR using primers listed in Table S1. NPC1^P692S^-GFP, FLOT2^Y124G/Y163G^-GFP, and FLOT2^Y124G/Y163G^-mCherry were generated with a site-directed mutagenesis kit (Stratagene, La Jolla, CA) from NPC1-GFP and FLOT2-mCherry plasmids, respectively (Table S1).

10.1128/mBio.02299-21.6 TABLE S1 Primer sequences for cloning NPC1 mutant and FLOT2 mutants into plasmids. Download Table S1, DOCX file, 0.02 MB. Copyright © 2021 Huang et al. 2021 Huang et al. https://creativecommons.org/licenses/by/4.0/ This content is distributed under the terms of the Creative Commons Attribution 4.0 International license.

### A. phagocytophilum and cell culture.

Cultivation of A. phagocytophilum HZ strain in HL-60 cells (ATCC, Manassas, VA), preparation of host cell-free A. phagocytophilum, and infection were performed as described previously (6). The degree of bacterial infection in host cells was assessed by Diff-Quik staining (Baxter Scientific Products, Obetz, OH), and the number of A. phagocytophilum cells was scored in 200 host cells in triplicate culture wells as described previously (40) or by quantitative PCR to detect the A. phagocytophilum *16S rRNA* gene normalized by the human *GAPDH* gene (6). RF/6A monkey endothelial cells (ATCC) were used in immunofluorescence labeling for unambiguous localization analysis owing to their tight adherence and thinly spread morphology (5). Human embryonic kidney HEK293T cells (ATCC) were used for transfection and pulldown assays, as they can be transfected with high efficiency (41).

### Transfection and immunofluorescence labeling.

RF/6A cells were cultured in advanced minimal essential medium (AMEM; Gibco, Waltham, MA) supplemented with 5% fetal bovine serum and 2 mM l-glutamine. Cells were transfected with plasmid NPC1-GFP, NPC1^P692S^-GFP, FLOT2-GFP, FLOT2-mCherry, HA-FLOT2^1–183^, FLOT2^1–183^-mCherry, FLOT2^Y124G/Y163G^-GFP, or FLOT2^Y124G/Y163G^-mCherry via electroporation (Gene Pulser Xcell; Bio-Rad, Hercules, CA). Subcellular localization of proteins in transfected cells was analyzed at 2 dpt. Cells were fixed in 4% PFA dissolved in Dulbecco’s phosphate-buffered saline (PBS [137 mM NaCl, 2.7 mM KCl, 10 mM Na_2_HPO_4_, 2 mM KH_2_PO_4_, pH 7.4]) at room temperature for 20 min and stained with 300 nM DAPI (4′,6′-diamidino-2-phenylindole; Invitrogen) in PGS buffer (PBS supplemented with 0.1% gelatin [Sigma, St. Louis, MO] and 0.3% saponin [Sigma]) for 15 min. For HA labeling, RF/6A cells cotransfected with NPC1-GFP and HA-FLOT2^1–183^ were infected with A. phagocytophilum at 4 hpt and treated with the membrane-permeable serine protease inhibitor diisopropylfluorophosphate (DFP) (Sigma) for 2 h prior to harvesting to inhibit bacterial surface serine protease (42). At 3 dpt, cells were fixed in 4% PFA at room temperature for 20 min and incubated with mouse monoclonal anti-HA followed by Alexa Fluor 555-conjugated goat anti-mouse IgG in PGS buffer.

For endogenous FLOT2 and NPC1 labeling, uninfected cells or A. phagocytophilum*-*infected cells treated with DFP for 2 h prior to harvesting were fixed in methanol-acetone (80:20) at –20°C for 10 min and incubated with mouse anti-FLOT2 and rabbit anti-NPC1 in PBS containing 0.1% gelatin for 1 h at 37°C, followed by incubation with Alexa Fluor 488-conjugated goat anti-rabbit IgG and Alexa Fluor 555-conjugated goat anti-mouse IgG for 30 min at room temperature.

### TF-cholesterol, DiI, and filipin labeling.

RF/6A cells were transfected with HA-FLOT2^1–183^, seeded onto glass coverslips in a 6-well plate, and cultured in AMEM supplemented with 5% fetal bovine serum and 2 mM l-glutamine at 37°C for 4 h prior to addition of host cell-free A. phagocytophilum. For TF-cholesterol (Avanti Polar Lipids, Alabaster, AL) labeling, infected RF/6A cells at 1 dpi were washed three times with serum-free AMEM, and the medium was replaced with AMEM supplemented with 5% lipoprotein-depleted serum (LPDS; Kalen Biomedical, Germantown, MD). After culturing for 8 h, TF-cholesterol (1 μM final concentration) was added to cells with subsequent incubation for 1 day (31). For cholesterol labeling with filipin (Sigma), RF/6A cells were fixed with 4% PFA for 15 min and stained with 50 μg/ml filipin in PBS at room temperature for 1 h and observed under a DeltaVision microscope.

For DiI and TF-cholesterol labeling with ezetimibe treatment, RF/6A cells were seeded onto a coverglass for 3 h and infected with A. phagocytophilum. At 1 dpi, cells were treated with dimethyl sulfoxide (DMSO) control or 40 μM ezetimibe for 14 h and then incubated with 5 μM Vybrant DiI cell-labeling solution (Thermo Fisher Scientific, Waltham, MA) for 6 h in the presence of ezetimibe. Alternatively, at 20 hpi, cells were incubated with 2 μM TF-cholesterol in AMEM containing 5% LPDS for 8 h and then treated with DMSO control or 40 μM ezetimibe for 20 h in the presence of TF-cholesterol. Cells were washed 3 times with PBS, fixed in 4% PFA for 15 min, and incubated with a cell-permeable DNA dye (1 μg/ml Hoechst 33342; Invitrogen) for 15 min to stain A. phagocytophilum and host DNA. The coverslip was mounted onto a slide using SlowFade Diamond antifade mountant (Invitrogen) and sealed with a coverslip sealant containing no organic solvents (Biotum, Fremont, CA).

### DeltaVision microscopy and image analysis.

Fluorescence and differential interference contrast images were captured with a DeltaVision personal DV deconvolution microscope system (GE Healthcare, Marlborough, MA). Data were processed using softWoRx (GE Healthcare) and Adobe Photoshop (Adobe Systems, Mountain View, CA). Colocalization was analyzed with softWoRx for the calculation of Pearson’s correlation coefficient. Colocalization of NPC1-GFP and FLOT2-mCherry in MβCD-treated or ezetimibe-treated cells was analyzed in 30 cells per group in each of three independent experiments. Colocalization of NPC1^P692S^-GFP and FLOT2-mCherry was analyzed in 30 cells per group in each of three independent experiments. Line-profile analyses were performed on a single *z*-section using ImageJ (NIH, Bethesda, MD), and relative signal intensity profiles were generated for FLOT, NPC1, and/or DAPI from selected vesicles and inclusions after normalizing values to the highest fluorescence intensity. For DiI and TF-cholesterol labeling, line-profile analyses and signal intensities of each A. phagocytophilum inclusion were performed on a single *z*-section using softWoRx. The intensities of the filipin signal in inclusions were measured in 30 ezetimibe-treated and 30 untreated cells using ImageJ.

### Immunoprecipitation and Western blotting.

HEK293T cells were suspended in the lysis buffer (150 mM NaCl, 25 mM Tris-HCl, pH 7.6, 1% [wt/vol] NP-40) containing freshly added protease inhibitor cocktail set III (CalBiochem, San Diego, CA) and incubated with mouse monoclonal anti-FLOT2 or normal mouse IgG on an end-to-end rotator at 4°C overnight. Protein G-agarose (Santa Cruz Biotechnology) was washed with ice-cold lysis buffer and incubated with the treated cell lysate on an end-to-end rotator at 4°C for 2 h. Transfected HEK293T cells were suspended in the lysis buffer containing freshly added protease inhibitor cocktail set III and incubated with anti-GFP nanobody affinity gel (BioLegend, San Diego, CA) on an end-to-end rotator at 4°C for 4 h. Beads were washed three times with ice-cold lysis buffer and then combined with 2× SDS sample buffer (4% [wt/vol] SDS, 135 mM Tris-HCl, pH 6.8, 10% [vol/vol] glycerol, 10% [vol/vol] β-mercaptoethanol, and 0.01% bromophenol blue). Samples were subjected to SDS-PAGE with 6% or 10% (wt/vol) polyacrylamide gels, and proteins were wet transferred (Bio-Rad) to a nitrocellulose membrane overnight. The membrane was blocked in a blocking buffer (5% [wt/vol] skim milk [Kroger, Cincinnati, OH], 150 mM NaCl, 50 mM Tris, pH 7.5) and then incubated with primary antibodies (1:1,000 dilution in blocking buffer) at 4°C for 12 to 16 h and subsequently with peroxidase-conjugated secondary antibodies (1:1,000 dilution) at room temperature for 1 h. Immunoreactive bands were visualized with enhanced chemiluminescence (Thermo Fisher Scientific) using a FujiFilm LAS3000 (FUJIFILM Medical Systems, Stamford, CT) or Amersham AI680QC gel documentation system (GE Healthcare), and band intensities were determined by densitometry using ImageJ.

### Chemical treatment.

MβCD (Sigma) and MβCD loaded with cholesterol (Sigma) were dissolved in AMEM, added to RF/6A or HEK293T cells at 2 or 3 dpt, respectively, and retained in the growth medium for 40 min prior to harvesting. Ezetimibe dissolved in DMSO was added to cells at 2 dpt for 20 h prior to harvesting, or to the A. phagocytophilum*-*infected cells at indicated times postinfection, and retained in the growth medium throughout the incubation period or removed at specific time points as indicated. Each reagent was removed by washing with PBS, and cells were fixed with 4% PFA. Alternatively, host cell-free A. phagocytophilum purified from infected HL-60 cells was pretreated with ezetimibe in growth medium without fetal bovine serum for 30 min at 37°C. Ezetimibe was washed out with growth medium, and the bacteria were used to infect host cells. The degree of bacterial infection in host cells was assessed as described above. Cell viability was examined by trypan blue (Sigma) exclusion test (43).

### Statistical analysis.

Statistical analysis was performed with the Student’s unpaired *t* test or analysis of variance (ANOVA), and a *P *value of <0.05 was considered statistically significant. All statistical analyses were performed using Prism 8 (GraphPad, La Jolla, CA).
