# Supplementary figures and images for: Anaplasma phagocytophilum Hijacks Flotillin and NPC1 Complex To Acquire Intracellular Cholesterol for Proliferation, Which Can Be Inhibited with Ezetimibe
Source: mBio. 2021 Sep 21;12(5):e02299-21. doi: 10.1128/mBio.02299-21 (PMC8546544; doi:10.1128/mBio.02299-21)

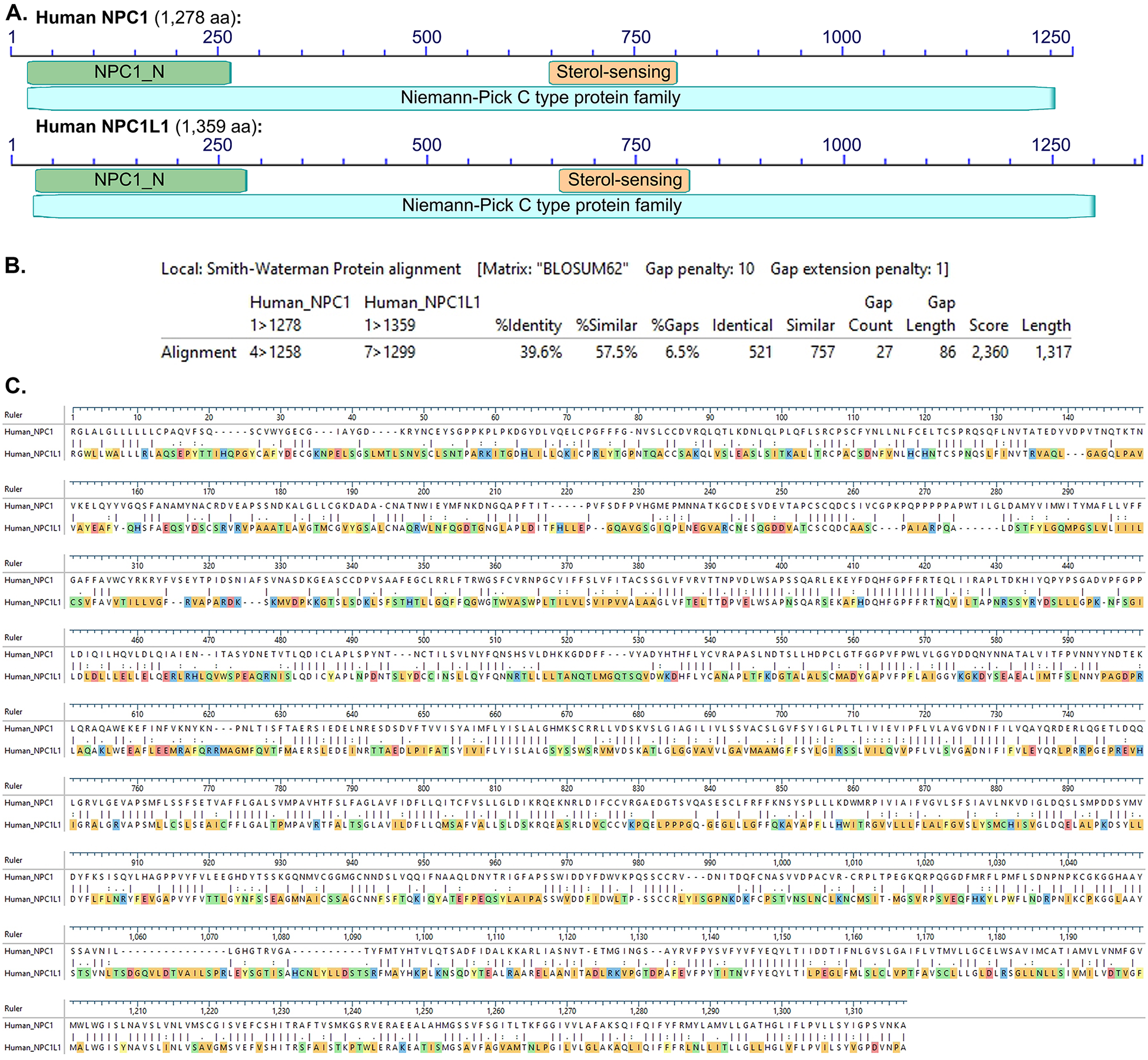

Supplement: FIG S1 [file mbio.02299-21-sf001.tif]

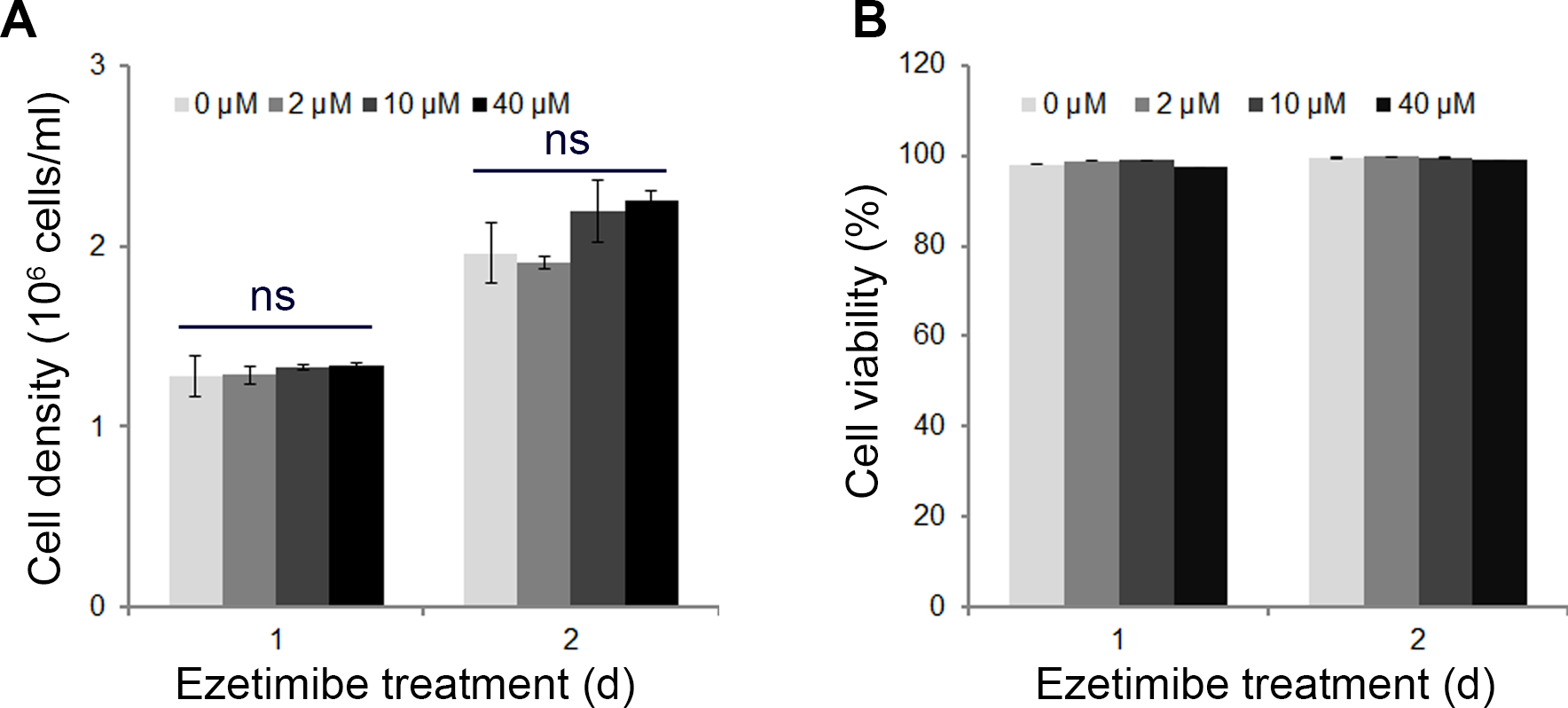

Supplement: FIG S2 [file mbio.02299-21-sf002.tif]

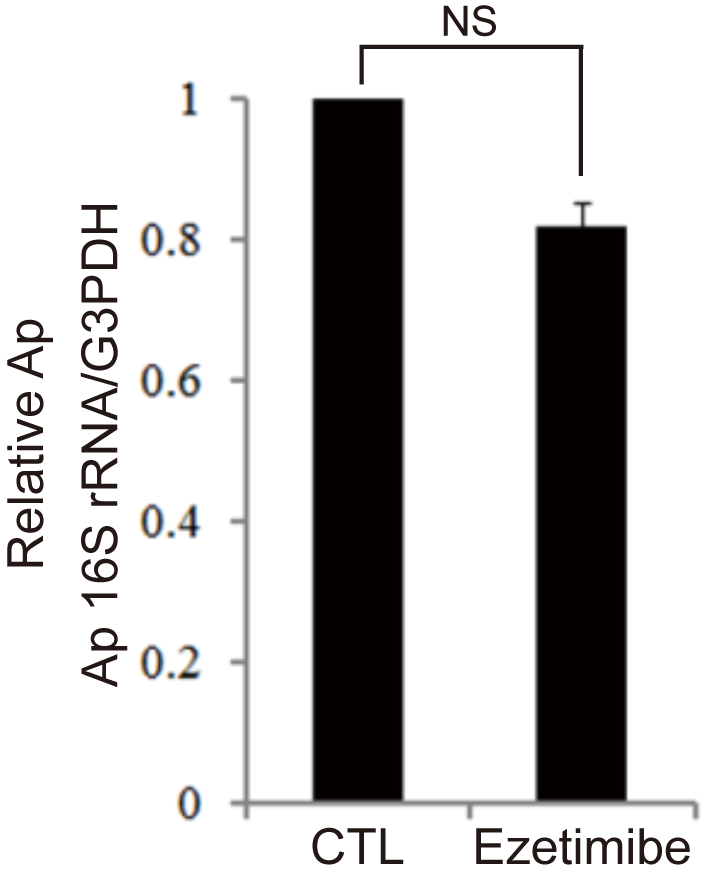

Supplement: FIG S3 [file mbio.02299-21-sf003.tif]

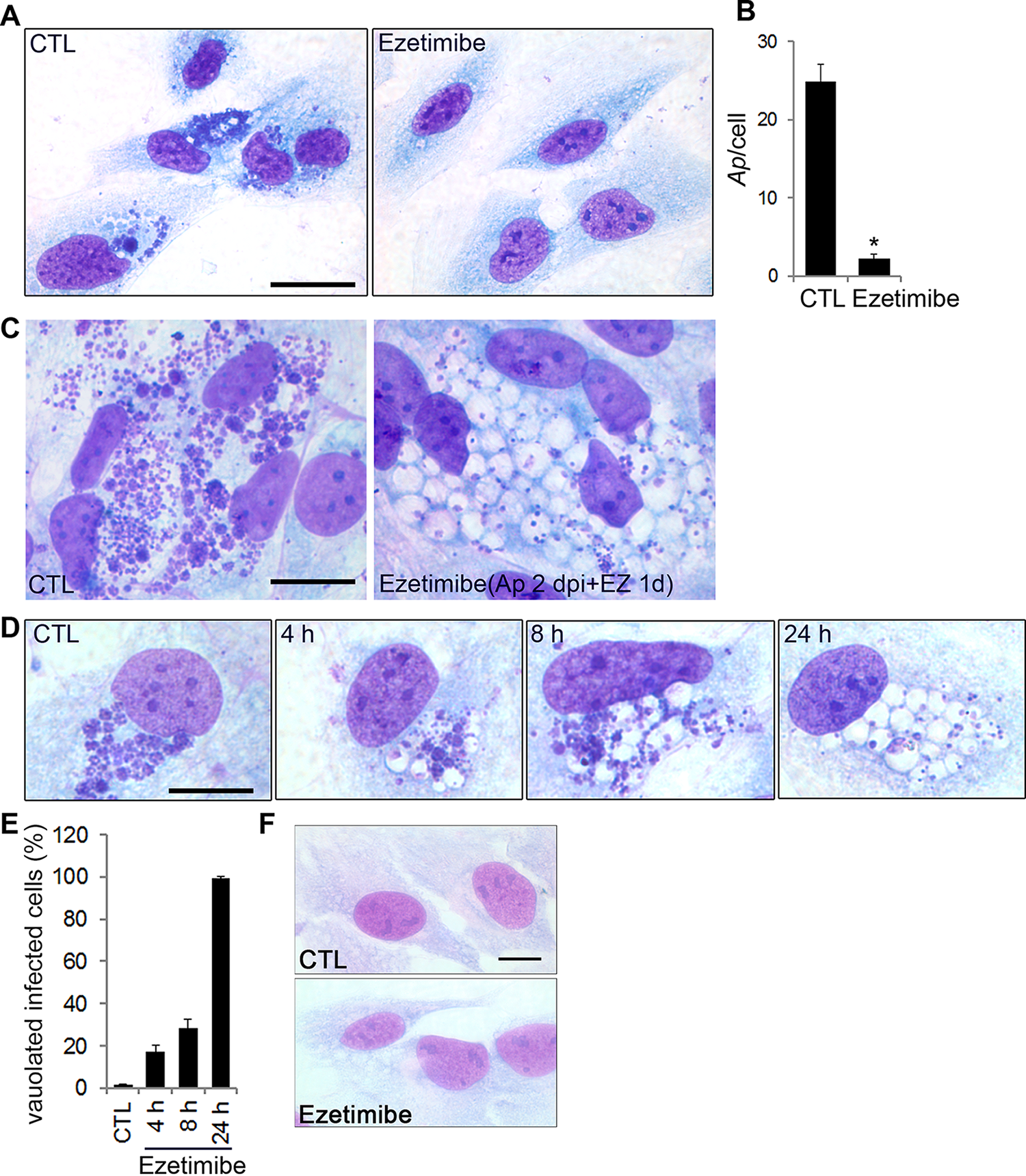

Supplement: FIG S4 [file mbio.02299-21-sf004.tif]

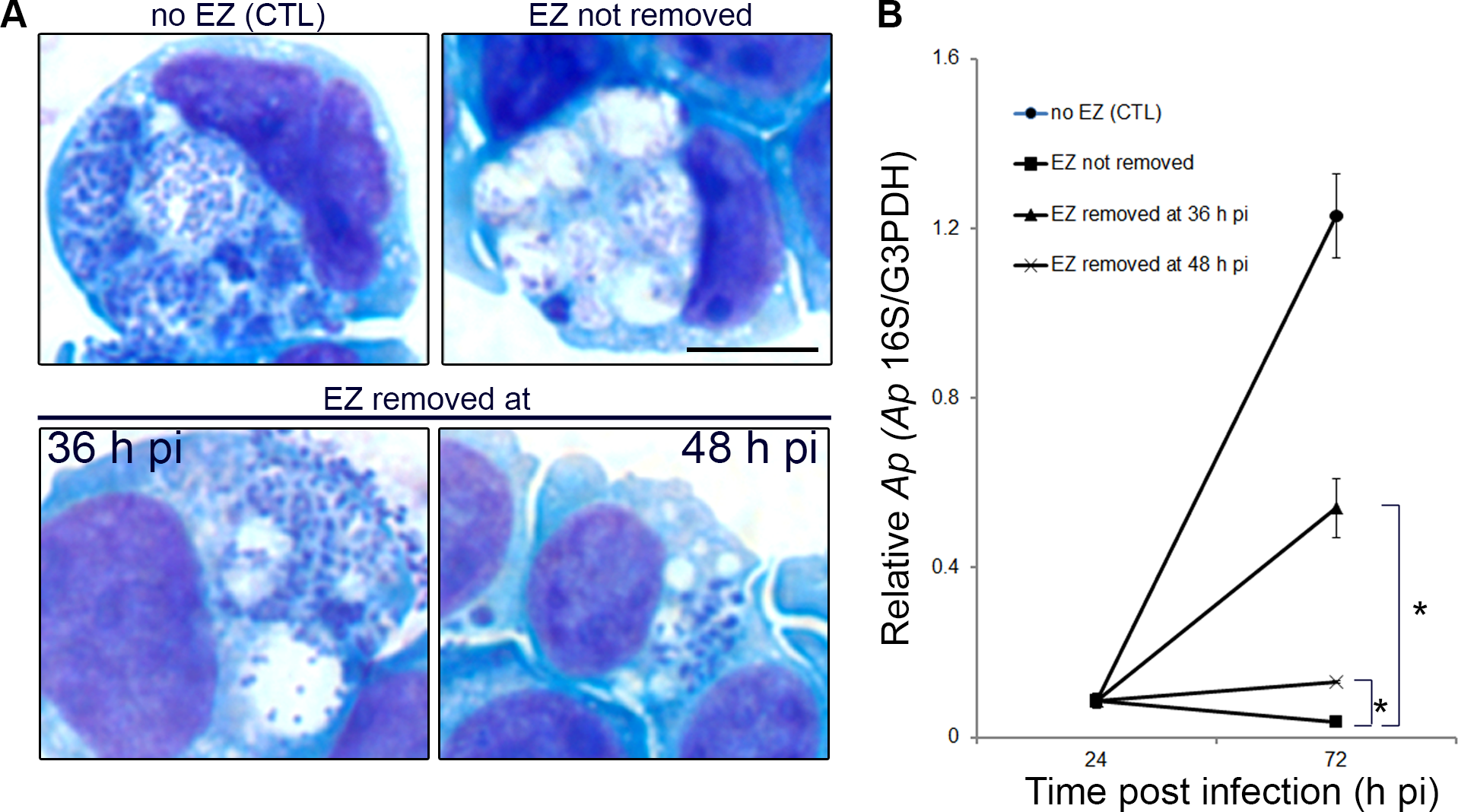

Supplement: FIG S5 [file mbio.02299-21-sf005.tif]
