# Supplementary material for: Anaplasma phagocytophilum Hijacks Flotillin and NPC1 Complex To Acquire Intracellular Cholesterol for Proliferation, Which Can Be Inhibited with Ezetimibe
Source: mBio. 2021 Sep 21;12(5):e02299-21. doi: 10.1128/mBio.02299-21 (PMC8546544; doi:10.1128/mBio.02299-21)
Supplement: TABLE S1 [file mbio.02299-21-s0001.docx]

## Table S1. Primer sequences for cloning NPC1 mutant and FLOT2 mutants into plasmids

| **Target** | **Primer sequence ^^[[1]](#footnote-1)^^** | **Note** |
| --- | --- | --- |
| NPC1^P692S^ | F: CTCATCGTCATTGAGGTCATC***TCA***TT CCTGGTGCTGGCTGTC  R: GACAGCCAGCACCAGGAA***TGA***GATG ACCTCAATGACGATGAG | For site-directed mutagenesis of Proline^692^ to Serine in SSD of NPC1. |
| FLOT2^1-183^-GFP | F: TACG CTCGAG *CGCCACCATGGGC* AATTGCCACACGGT  R: CGTA GGATCC CGCACGCCAATGT CAGCATCTCT | F, XhoI site; R, BamHI. For cloning FLOT2^1-183^ into XhoI (F) and BamHI (R) sites on EGFP-N1 plasmid |
| FLOT2^Y124G^-mCherry | F: ACCCTGACAGTGGAGCAGATT***GGT***CA GGACCGGGACCAGTTTGCC  R: GGCAAACTGGTCCCGGTCCTG***ACC***AA TCTGCTCCACTGTCAGGGT | For first step site-directed mutagenesis of Tyrosine^124^ to glycine in cholesterol binding domain in FLOT2-mCherry. |
| FLOT2^Y163G^-mCherry | F: GACGTGTATGACAAAGTGGAC***GGT***CT GAGCTC CCTGGGCAAGACG  R: CGTCTTGCCCAGGGAGCTCAG***ACC***G TCCACTT TGTCATACACGTC | For second step site-directed mutagenesis of Tyrosine^163^ to glycine in cholesterol binding domain in FLOT2-mCherry. |
| HA-FLOT2^1–183^ | F1: *GTTCCAGATTACGCT*GGCAATTGCC ACACGGTGGGGC  F2: GCATCTCGAGCGCCACCATG*TACCC* *ATACGATGTTCCAGATTACGCT*GGC  R: AGTGCGGCCGCTCACACGCCAATGT CAGCATC | For cloning HA-FLOT2^1-183^ into XhoI (F2) and NotI (R) sites on pEGFP-N1 plasmid.  Sequence-coding HA-tag is italicized, which is attached to the N-terminal FLOT2 sequences through two-step PCR amplifications using overlapping forward primers (first-step: F1+R; second-step: F2+R). |

1. F, forward; R, reverse complement primers; underlined sequences, restriction enzyme sites; italicized, Kozak sequences for mammalian expression in pEGFP-N1 plasmid; Bold and italicized sequences: codon sequences for site-directed mutagenesis. [↑](#footnote-ref-1)
